# DOTA Glycodendrimers as Cu(II) Complexing Agents and Their Dynamic Interaction Characteristics toward Liposomes

**DOI:** 10.1021/acs.langmuir.0c01776

**Published:** 2020-09-30

**Authors:** Marianna Carone, Silvia Moreno, Michela Cangiotti, Maria Francesca Ottaviani, Peng Wang, Riccardo Carloni, Dietmar Appelhans

**Affiliations:** † Department of Chemistry and Biochemistry, University of Bern, 3012 Bern, Switzerland; ‡ Leibniz Institute of Polymer Research Dresden, Hohe Strasse 6, D-01069 Dresden, Germany; § Department of Pure and Applied Sciences, Università degli studi di Urbino “Carlo Bo”, Urbino 61029, Italy

## Abstract

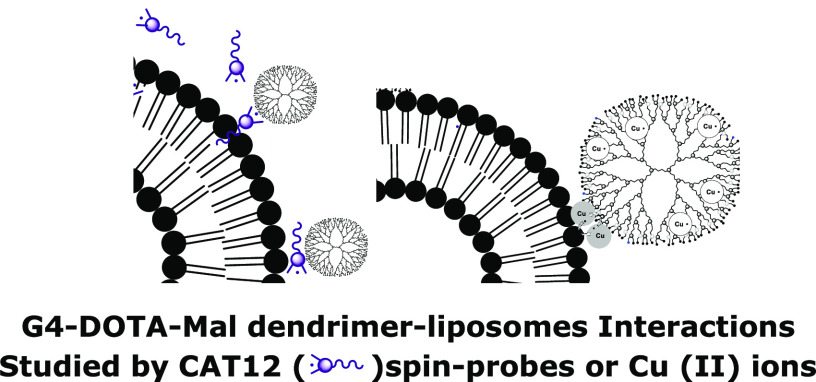

Copper (Cu)(II) ions, mainly an excess amount, play a negative role in the course of several diseases, like cancers, neurodegenerative diseases, and the so-called Wilson disease. On the contrary, Cu(II) ions are also capable of improving anticancer drug efficiency. For this reason, it is of great interest to study the interacting ability of Cu(II)–nanodrug and Cu(II)–nanocarrier complexes with cell membranes for their potential use as nanotherapeutics. In this study, the complex interaction between 1,4,7,10-tetraazacyclododecan-N,N′,N′′,N′′′-tetraacetic acid (DOTA)-functionalized poly(propyleneimine) (PPI) glycodendrimers and Cu(II) ions and/or neutral and anionic lipid membrane models using different liposomes is described. These interactions were investigated *via* dynamic light scattering (DLS), ζ-potential (ZP), electron paramagnetic resonance (EPR), fluorescence anisotropy, and cryogenic transmission electron microscopy (cryo-TEM). Structural and dynamic information about the PPI glycodendrimer and its Cu(II) complexes toward liposomes was obtained *via* EPR. At the binding site Cu–N_2_O_2_ coordination prevails, while at the external interface, this coordination partially weakens due to competitive dendrimer–liposome interactions, with only small liposome structural perturbation. Fluorescence anisotropy was used to evaluate the membrane fluidity of both the hydrophobic and hydrophilic parts of the lipid bilayer, while DLS and ZP allowed us to determine the distribution profile of the nanoparticle (PPI glycodendrimer and liposomes) size and surface charge, respectively. From this multitechnique approach, it is deduced that DOTA-PPI glycodendrimers selectively extract Cu(II) ions from the bioenvironment, while these complexes interact with the liposome surface, preferentially with even more negatively charged liposomes. However, these complexes are not able to cross the cell membrane model and poorly perturb the membrane structure, showing their potential for biomedical use.

## Introduction

Copper is an essential microelement for the body whose intake comes from diet.^1^ A very sophisticated system ensures safe transportation to the sites of interest and safe elimination through biliary secretion.^2^ However, Cu(II) ions may play a toxic role in several diseases, like Alzheimer’s disease, cancer, and, more specifically, the so-called Wilson disease (WD). WD is a rare disease that affects copper transportation, provoking the accumulation of copper ions in the liver.^3−11^ Not only this increasing copper deposition eventually compromises hepatic function but also, when the hepatic storage capacity is exceeded, the unbound copper ions are poured from the liver to the bloodstream and deposited in other organs and tissues, leading, among the others, to neurological and psychiatric complications.

Medical treatments for avoiding the toxic effects of an excess of Cu(II) ions are mainly limited to the use of low-molecular-weight chelating agents, such as d-penicillamine and Trientine,^7,12^ which are associated with numerous side effects, including neurological deterioration, sideroblastic anemia, and hypersensitive reactions.^7^ Under this shadow, nanotechnology offers an unprecedented opportunity to tailor drugs with a view to find a new class of chelators for copper ions, as an alternative to penicillamine. Several efforts have been made in the direction of creating highly biocompatible nanosized macromolecules, which can be used as nanocarriers and *per se* as polymeric therapeutics.^13,14^ Very promising nanoparticles in this field are dendrimers. Dendrimers are perfectly branched, nanometer-sized, and monodisperse structures with a strictly tailored architecture consisting of a central core surrounded by repeating layers (termed generations) of chemical units. The external end groups can be functionalized for optimizing their pharmacokinetics or biological properties.^15^ Despite the many advantages of these nanoparticles, previous research studies have demonstrated that unmodified amino-terminated dendrimers are not ideal candidates for medical applications due to their high cytotoxicity.^16^ Specifically, the toxicity comes from the strong electrostatic interaction, which is established between the positively charged dendrimers and negatively charged cell membranes, leading to cell lysis. This behavior is the main reason why cationic dendrimers are rapidly cleared from plasma.^17^ Therefore, modification of the periphery may be used for adapting cellular interactions and biodistribution of dendrimers. Additionally, the trigger or inhibition of biological events is usually determined by carbohydrate–protein interactions such as immune response^18,19^ or bacterial adhesion.^20^ Under this shadow, the combination of carbohydrate and dendrimers not only reduces the toxicity but also improves the biocompatibility, thus allowing glycodendrimers to be used as polymeric therapeutics and diagnostics for *in vitro* and *in vivo* studies.^13,21−29^ For example, poly(propyleneimine) (PPI) glycodendrimers are suited materials with promising potential as antiamyloidogenic agents for treating and hampering undesired peptide/protein aggregation in neurodegenerative diseases (Alzheimer’s disease, prion disease, and sporadic Jakob–Creutzfeldt disease). They also show general neuroprotective properties for improving memory and synapses function by crossing the blood–brain barrier.^21−23^

**Figure 1:**
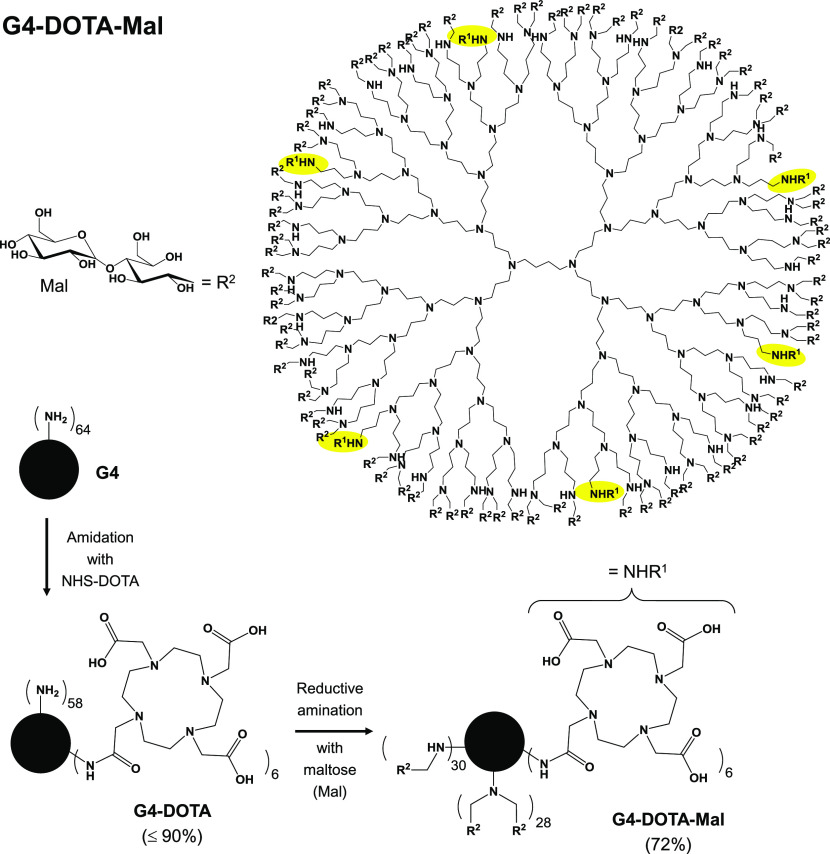
Simplified structure of G4-DOTA-Mal attributed by total functionalization of terminal NH_2_ groups with maltose and six DOTA groups. The synthetic route is also shown.

The aim of this study was to find new dendrimeric structures able to complex with an excess of Cu(II) ions. This includes the characterization of their resulting Cu(II) complexes as well as their interactions with biostructures, starting from simplified cell membrane models. Motivated by our previous studies,^13,21−23,26^ a fourth-generation PPI glycodendrimer, constituted by a dense maltose (Mal) shell, was enriched with six groups of 1,4,7,10-tetraazacyclododecan-N,N′,N′′,N′′′-tetraacetic acid (DOTA), presenting specific chelating properties for copper ions and a few other rare metals, including gadolinium.^27^ For simplicity, this PPI glycodendrimer (Figure 1) was termed as G4-DOTA-Mal.

On the basis of previous studies dealing with the interactions of dendrimers with biological membranes,^28−32^ liposomes were used as simplified cell membrane models to investigate their interactions with the G4-DOTA-Mal dendrimer and its copper complexes. Thus, it was possible to imitate the impact of G4-DOTA-Mal and its Cu(II) complexes on cell membrane fluidity, permeability, and fusion. The use of membrane models like liposomes was justified because (i) the physicochemical interaction study is not affected by the influence of membrane or plasma proteins, (ii) the liposomes are characterized by high reproducibility and long-term stability, (iii) we used phospholipids that are naturally present in biological membranes. To simulate dendrimer–cell interactions, different membrane models were used, specifically, liposomes constituted by egg lecithin (termed LEC), 1,2-dimyristoyl-*sn*-glycero-3-phosphocholine (termed as DMPC), and a binary mixture of DMPC and 1,2-dimyristoyl-*sn*-glycero-3-phospho-rac-(1-glycerol) sodium salt (DMPG, 3%). In the last case, the liposomes were simply termed as DMPG.

We selected lecithin since we recently verified its suitability as model membranes to analyze interactions with dendrimers.^32^ On the other side, DMPC has been found to satisfactorily work as a model membrane interacting with dendrimers.^28,29^ The addition of 3% DMPG is enough to change the interacting ability of the liposome surface and the structural properties as tested by the variations of the parameters extracted by means of the different techniques used in the present study. Indeed, the interacting ability of the G4-DOTA-Mal dendrimer with the above-mentioned membrane models was investigated through different experimental techniques: dynamic light scattering (DLS) and ζ-potential (ZP) that allowed us to determine the distribution profile of the nanoparticle size and surface charge, respectively; fluorescence anisotropy, which is used to evaluate the membrane fluidity of both the hydrophobic and hydrophilic parts of the lipid bilayer;^33^ and cryogenic transmission electron microscopy (cryo-TEM) and electron paramagnetic resonance (EPR), which provided structural and dynamic information about the dendrimer–liposome systems and characterized their interacting behavior, respectively. Based on the previous studies, two different approaches were followed in the EPR investigation: (i) analyzing selected surfactant radicals, inserted in the membrane models,^28−31^ and (ii) focusing on Cu(II) ions as a complexing agent,^32,34−42^ measuring the interacting strength between the coordinated dendrimers and the membrane models. This multitechnique study allowed us to verify that the G4-DOTA-Mal glycodendrimer is a good and selective Cu(II) complexing agent and how these G4-DOTA-Mal glycodendrimer–Cu(II) complexes interact with lipid bilayers predicting the phenomena that may happen in the presence of biological membranes.

## Experimental Section

### Materials

The phospholipids—1,2-diacyl-*sn*-glycero-3-phosphocholine (egg lecithin), 1,2-dimyristoyl-*sn*-glycero-3-phosphocholine (DMPC), and 1,2-dimyristoyl-*sn*-glycero-3-phospho-rac-(1-glycerol) sodium salt (DMPG)—, phosphate-buffered saline (PBS), copper(II) nitrate hydrate, and fluorescent probes—1,6-diphenyl-1,3,5-hexatriene (DPH) and *N*,*N*,*N*-trimethyl-4-(6-phenyl-1,3,5-hexatrien-1-yl)phenylammonium *p*-toluenesulfonate (TMA-DPH)—were purchased from Merck (Germany). PPI dendrimer was supplied by SyMoChem (Eindhoven, The Netherlands). The synthesis and characterization of G4-DOTA-Mal is described in the Supporting Information (SI), following a previously published procedure.^43^ The nomenclature of PPI dendrimers is based on Tomalia et al.^44^ CAT12 surfactant nitroxide radical (4-(dodecyl dimethyl ammonium)-1-oxyl-2,2,6,6-tetramethyl piperidine bromide) was supplied by Chemical Laboratories of Columbia University (New York).

### Liposome Preparation in the Absence and Presence of G4-DOTA-Mal

Three different liposomes were prepared: (i) lecithin liposomes (termed as LEC), (ii) DMPC liposomes (termed as DMPC), and (iii) DMPC/DMPG 3% liposomes (termed as DMPG). In all cases, the following preparation protocol is followed. Phospholipids were dissolved under stirring in 2:1 chloroform/methanol to obtain a 0.65 mM solution for the DLS, cryo-TEM, ZP, and fluorescence measurements and a 25 mM solution for EPR measurements. Then, the organic solvents were evaporated under vacuum at 40 °C using a rotary evaporator (Rotavapor). The obtained lipid thin film was placed under vacuum for 24 h and then hydrated with a PBS (10 mM, pH 7.4) solution in the absence and presence of G4-DOTA-Mal at a concentration of 0.05 mM for DLS, cryo-TEM, ZP, and fluorescence measurements and 1.56 mM for EPR measurements (corresponding to 0.2 M in external surface groups). The higher concentrations for EPR measurements were needed due to the sensitivity of the technique. However, the same molar ratio of the phospholipid to the dendrimer (16) was used for all techniques. The PBS solution was selected as the solvent for two reasons: (i) to maintain pH in neutral conditions to better compare the experimental results from various samples and various techniques and (ii) to use a salt solution as used with cell cultures. However, the presence of PBS affects the ZP, as well as the interacting ability of liposomes and dendrimers, but we are interested in investigating the interacting behavior mimicking the biological conditions. Hydration was carried out under mechanical stirring for 1 h at 40 °C.^45^ The sample thus obtained was divided into two parts, and each part was subjected to sonication or extrusion treatment as described in the following: (i) sonication method: 10 cycles (30 s interspersed + 30 s pause) and the resulting suspension containing liposomes was incubated at 40 °C overnight (*C*_F_ = 25 mM); (ii) extrusion method: the suspension was extruded through a polycarbonate filter (100 nm pore size filter, 11 times) with two 1000 μL Hamilton gastight syringes at 40 °C using an Avant Mini extruder (*C*_F_ = 25 mM) and the suspension thus obtained was incubated at 40 °C overnight (*C*_F_ = 25 mM).

A study was first performed to verify the stability of liposomes obtained by means of two methods. On the basis of these results, for the characterization of the interactions with the dendrimer, only the extrusion method was used since the liposomes demonstrated long-time structural stability.

### EPR, DLS, ZP, Cryo-TEM, and Fluorescence Anisotropy Experiments

Experimental descriptions of these methods are presented in the SI along with the concentrations of liposomes, G4-DOTA-Mal, CAT12, and Cu(II).

## Results and Discussion

### Characterization of Liposomes, G4-DOTA-Mal, and Their Mixtures in the Absence and Presence of Cu(II) Using DLS, ZP, and Cryo-TEM

Prerequisites for the validation of interaction characteristics of G4-DOTA-Mal/Cu(II) complexes in the presence of liposome models by the EPR study were to first examine the properties (size and charge) and the stability of liposomes (LEC, DMPC, and DMPG) in the presence of G4-DOTA-Mal, followed by the addition of increasing Cu(II) concentrations at 0, 1, and 24 h, using DLS and ZP. This also includes the use of cryo-TEM for visualizing and comparing liposomes in the absence and presence of G4-DOTA-Mal. Stable and well-characterized vesicles are needed to perform EPR and fluorescence anisotropy experiments. The dynamics of liposomes (*e.g*, fusion and fission) *per se* need to be measured to get the right conclusions for the G4-DOTA-Mal–liposome interactions in the presence and absence of Cu(II).

Table S1 (SI) summarizes the hydrodynamic diameters (*D*_h_, *z*-average) and surface charge (ζ) of G4-DOTA-Mal (0.05 M) in the absence and presence of Cu(II) (7, 14, and 28 mM) at 25 and 37 °C, respectively. In comparison to nonaggregated G4-DOTA-Mal macromolecules in aqueous solutions (Figure S3), G4-DOTA-Mal macromolecules immediately aggregate in the PBS solution (10 mM) (337 nm at 25 °C and 670 nm at 37 °C, Table S1 in the SI). This indicates the action of phosphate ions as a gluing agent in PBS-containing dendrimer solutions at which uncontrolled aggregation of G4-DOTA-Mal occurs, justified by the high polydispersity index (PDI) values (Table S1) at both applied temperatures. The same situation occurs when Cu(II) is added, still showing high PDI values (from 0.7 for 7 mM Cu(II) to 0.4 for 28 mM Cu(II)) due to aggregated Cu(II)/G4-DOTA-Mal complexes with higher and lower *z*-average data. This demonstrates that the gluing properties of phosphate ions between G4-DOTA-Mal macromolecules are partially diminished by the highest Cu(II) concentration. The addition of Cu(II) also provokes an increase in the cationic surface charge (ζ) of G4-DOTA-Mal aggregates (from 16.5 to 32 mV at 25 °C). The EPR study (see below) demonstrates that this kind of glycodendrimer is able to complex Cu(II). Overall, G4-DOTA-Mal PBS solutions are highly polydispersive due to the presence of dendrimer aggregates in the absence and presence of Cu(II) tailored by noncovalent interactions.

Table S2 (SI) depicts the hydrodynamic diameters (*D*_h_, *z*-average) and surface charge (ζ) of liposomes (LEC, DMPC, and DMPG) in the absence and presence of G4-DOTA-Mal and their adducts with Cu(II) at three concentrations (7, 14, and 28 mM), determined at 0, 1, and 24 h. The almost invariance of the size over time of the two- and three-component systems—liposome/G4-DOTA-Mal and liposome/G4-DOTA-Mal/Cu(II)—is proved by DLS experiments, which show the stability of each liposome system at different compositions after 24 h. However, the PDI values change from 0.43 to 0.15 with still partly high values after 24 h. On the basis of the variations of *D*_h_ (*z*-average data) and ζ in Table S2 for liposomes in the presence of G4-DOTA-Mal and various Cu(II) concentrations extracted from DLS and ZP results after 24 h at 25 °C, we note that (i) all liposomes show similar *D*_h_ values (⌀ 120–130 nm); (ii) the addition of G4-DOTA-Mal to all liposomes provides a slight increase or decrease of *D*_h_ (±≤10 nm), implying that each liposome is stable in the presence of G4-DOTA-Mal; and (iii) by further adding Cu(II) at increasing concentrations, the liposome diameter, after an evident instability at time 0, already after 1 h, and, more, at 24 h, changes only slightly, underlining the desired stability of liposomes (±≤10 nm compared to pure liposomes with ⌀ 120–130 nm). Two exceptions are given by DMPC and DMPG with G4-DOTA-Mal and 28 mM Cu(II), showing *D*_h_ values of about 170 and 96 nm, respectively. The size increase may be related to the formation of adducts driven by the excess amount of Cu(II). Further details on the interacting behavior within the two- and three-component systems will be shown in the following EPR study part.

In any case, the small variations of the structural parameters in Table S2 for the liposomes from the absence to the presence of the dendrimers or dendrimer–Cu(II) complexes indicate that noncovalent interactions (electrostatic, dipole–dipole, H-bonds) in the two- and three-component systems exist to destroy or breakdown the undesired dendrimer and dendrimer/Cu(II) aggregates. This undoubtedly indicates the interfering properties of liposomes toward G4-DOTA-Mal macromolecules in PBS (10 mM) solutions (Table S2). Furthermore, isolated G4-DOTA-Mal and a few aggregated liposome/G4-DOTA-Mal hybrid structures in the absence and presence of Cu(II) are also assumed when considering the still high PDI values for two- and three-component systems with DMPC and DMPG. In this context, LEC systems slightly look more homogeneous than DMPC and DMPG systems. This further implies that the gluing properties of phosphate ions do not play any deciding role as in the case of one- and two-component systems of G4-DOTA-Mal and G4-DOTA-Mal/Cu(II) (Table S1).

Besides the achievement of *z*-average data by DLS (Table S2), a deeper analysis of the volume plots for liposomes in the presence of G4-DOTA-Mal and one selected Cu(II) concentration of 14 mM (Figure S5 in SI) further underlines the high stability of liposomes, outlining peak maxima for *D*_h_ at around 100 nm for DMPC and DMPG liposomes and a little bit higher (about 120 nm) for LEC liposomes. We observe a similar solution behavior of G4-DOTA-Mal macromolecules (Figure S5A) in the presence and absence of liposomes and Cu(II) to that discussed above. This thoroughly means that G4-DOTA-Mal macromolecules preferentially interact with the liposome surface due to the absence of any other particles in the volume plots (presenting three repeating measurements of each solution). Moreover, the Cu(II) complexation and/or interaction must occur in the environment of liposome/G4-DOTA-Mal hybrid structures.

The high stability of liposomes was further confirmed by long-term analysis over 30 days, storage at 4 and 25 °C, and use of different pure liposome concentrations (Figures S7 and S8 in SI). In all time ranges and different experimental conditions, the particle size and volume remain almost the same at 100 nm. Moreover, we expected that the surface charge of liposomes might be affected when pure G4-DOTA-Mal or the combination of G4-DOTA-Mal/Cu(II) is added to the liposome solutions. The DMPC surface is almost neutral, while, as expected, the DMPG surface is negatively charged. Conversely, the negative surface charge of the zwitterionic LEC liposome surface is not expected since LEC and DMPC have similar charges in the headgroups. However, the different packing of phosphatidylcholine (PC) and phosphatidylethanolamine (PE) lipids in LEC modulates the surface charge at the interface. By adding the cationic dendrimer macromolecules, first, and then different cationic Cu(II) concentrations, the surface charge differently increases but to a low extent (ZP in Table S2, SI). Surprisingly, there is no real change in the surface charge of liposome DMPC when adding G4-DOTA-Mal. This behavior may suggest weak interactions between the components. In the case of LEC and DMPG, a conversion from anionic to slightly positive surface charge is observed, leading to compensation at the highest Cu(II) concentration (28 mM). This may imply a stronger physical interaction at the interface of the liposome and dendrimer macromolecule in the absence and presence of Cu(II) compared to DMPC samples. The interactions within the two- and three-component systems (liposome/G4-DOTA-Mal and liposome/G4-DOTA-Mal/Cu(II)) are better characterized by the EPR and fluorescence analyses and are described below.

Finally, originally observed aggregates for G4-DOTA-Mal with/without Cu(II) (Table S1) are immediately destroyed or cannot be formed in the presence of liposomes. This event is confirmed by the observation using DLS, of much more disperse and heterogeneous solutions (Table S2).

**Figure 2:**
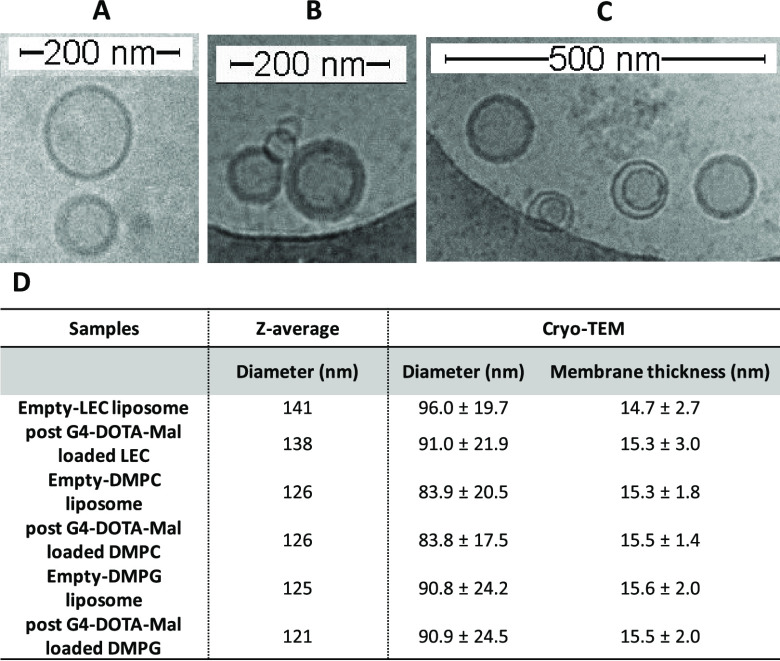
Cryo-TEM images of postloaded G4-DOTA-Mal with liposomes (A) LEC, (B) DMPC, and (C) DMPG. (D) Comparison of the data evaluated by DLS and cryo-TEM study. The used concentrations are reported in Section S4 in the SI.

To further underline the presence of stable liposomes with a unilamellar lipid bilayer structure, fabricated by the extrusion method, a cryo-TEM investigation was used to obtain additional information about the real diameter and membrane thickness since DLS did not provide desired characteristics regarding the morphology and shape of liposomes. Figure 2 highlights the comparison of pure and G4-DOTA-Mal-loaded liposome solutions (LEC, DMPC, and DMPG) determined by DLS and cryo-TEM in which defined concentrations of the components have been used. As known, the *D*_h_ from DLS results is always larger compared to diameters obtained from the analyzed cryo-TEM images.^46,47^ It is important to underline that the membrane thickness usually ranges between 6 and 4 nm, depending on the temperature. In our case, the thickness is much higher. However, it is reported in the literature that the effect of a soluble salt is a “threefold increased size as compared to single lipids” due to ions and water coordination at the charged lipid heads.^48^ First, the cryo-TEM study shows that the extrusion method is suited to fabricate unilamellar liposomes. This is a further prerequisite for the EPR study. Second, the postloading of G4-DOTA-Mal to liposome solutions does not show any effect on the diameter and membrane thickness of each liposome. Therefore, the model membrane does not integrate G4-DOTA-Mal after postloading. This certainly occurs for DMPC and DMPG, which do not show any structural variations after the addition of the dendrimer. Conversely, a small change in the liposome diameter is detected in LEC liposomes from the absence to the presence of G4-DOTA-Mal. The diameter slightly decreases (from 96 to 91 nm), while the membrane thickness keeps nearly constant. This open issue of the type and strength of interaction of G4-DOTA-Mal with liposome structures will be clarified hereafter by EPR and fluorescence anisotropy study. DLS, ZP, and cryo-TEM studies show that the presence of G4-DOTA-Mal does not affect the main structural characteristics of liposomes.

### EPR Study of the Interactions between DOTA Glycodendrimers Complexed by Cu(II) and Membrane Models (LEC, DMPC, and DMPG)

Two requirements are considered for the EPR analysis to emphasize the desired interaction characteristics between the G4-DOTA-Mal dendrimer and the liposomes (LEC, DMPC, and DMPG). The first requirement is achieved by adding a surfactant nitroxide radical (CAT12) at a concentration of 1 mM, which is lower than the CAT12 critical micelle concentration (cmc) value (7.1 mM at 295 K).^41^ We already found CAT12 suitable for analyzing liposome structures in previous studies, where the interactions between dendrimers and liposomes are studied in the absence and presence of Cu(II).^32^ The presence of an interacting component indicates that the probe enters the liposome, and we hypothesize that it mimics lipids in the liposome structure. A limited impact of CAT12 on the liposome structure was demonstrated by invariance in the EPR spectrum line shape by changing the CAT12 concentration up to 1 mM. The second requirement is to understand the complexation characteristics of G4-DOTA-Mal (1.56 mM) toward Cu(II) in the absence of liposomes by analyzing the EPR spectra of Cu(II) at different molar ratios with respect to the DOTA groups on the dendrimer surface (1:0.75/1:1.5/1:3 of DOTA/Cu).

#### Characterization of Liposomes and Their Interactions with G4-DOTA-Mal Using a Surfactant Nitroxide Radical

CAT12 is selected for this study since it has already proved to be a very informative spin probe for monitoring and validating structural modifications of liposomes and their membrane interactions with various dendrimers.^26^ Furthermore, CAT12 mimics the behavior of the phospholipids constituting the membrane and is able to insert into the lipid aggregates with the hydrophobic chain, while the paramagnetic CAT group remains at the liposome/water interface.

**Figure 3:**
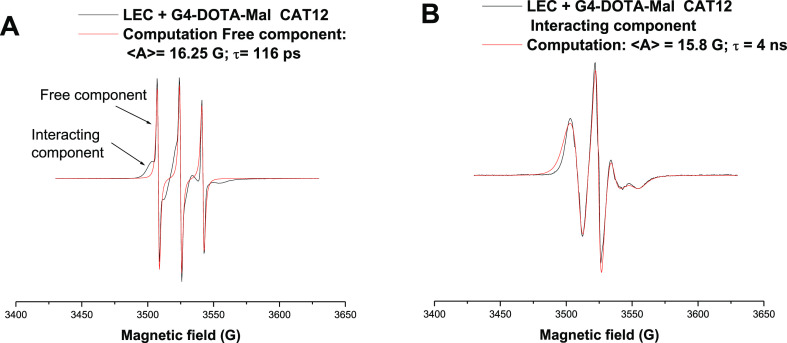
(A) EPR experimental spectra of CAT12 in the systems LEC + G4-DOTA-Mal, which also shows the computation of the free component. (B) Computation of the interacting component obtained after subtraction of the free component. The legends show the main parameters of computations.

**Scheme 1:**
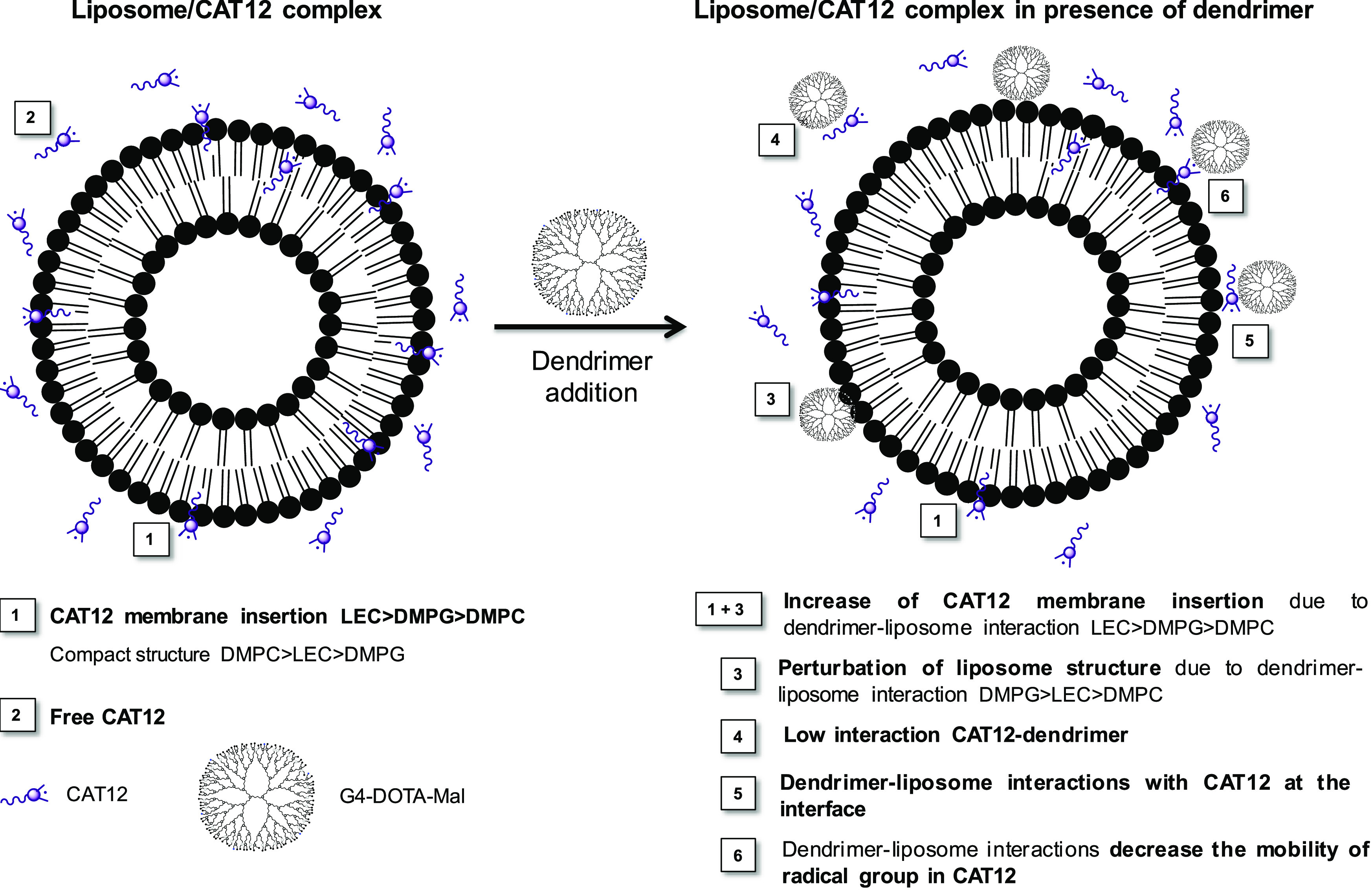
Different Distributions and Interactions of the Surfactant Radical, CAT12, Mimicking the Lipid Surfactants in the Liposome Membrane in the Absence and Presence of the G4-DOTA-Mal Dendrimer

Figure 3 shows the selected example for the experimental spectrum of CAT12 (1 mM) in the presence of the LEC (25 mM in phospholipids) + G4-DOTA-Mal (1.56 mM) system (A). The spectra were recorded at 25 °C. Experiments at 37 °C were also performed, and the results showed the same trends for the different samples but lower differences among the samples with respect to the experiments at 25 °C. Since we are mainly interested in the interacting behavior also differentiating the various samples, here, only the results at 25 °C are described. Two spectral components are identified, whose first peaks are indicated by arrows in Figure 3A. These components are termed as “free component” and “interacting component” on the basis of the line shape features. These two components are present also for the liposomes/CAT12 systems in the absence of the dendrimers (see, for example, Figure S9 in the SI). Indeed, the free component consists of three narrow lines. This is characteristic of radical groups that are free to rotate in the solution. The free component of CAT12 is present in all cases and is the only component present for the dendrimer in the absence of liposomes (Figure S10 in the SI). Figure 3A shows the computation of this free component for the case of LEC + G4-DOTA-Mal. Here, the well-known calculation method of Budil et al. (NLSL program)^49^ is used to obtain structural and dynamic parameters of CAT12 in the presence of G4-DOTA-Mal, Cu(II), and/or liposomes. The resulting main parameters, ⟨*A*⟩ and τ, are useful for the present study and are generally shown in figure legends: ⟨*A*⟩ is the hyperfine coupling constant between the electron spin and the nitrogen nuclear spin, expressed as ⟨*A*⟩ = (*A_xx_* + *A_yy_* + *A_zz_*)/3, ⟨*A*⟩ measures the micropolarity in the CAT12 environment, and τ is the correlation time for the rotational motion of CAT12, which measures the microviscosity and, consequently, the interaction strength of the CAT12 probe in the liposome membranes in the absence and presence of dendrimers. The values of ⟨*A*⟩ = 16.25 G and τ = 116 ps, obtained by simulating the free component in Figure 3A, indicate a fast-moving CAT12 radical group in the presence of liposomes LEC and G4-DOTA-Mal at the external interface. Considering the aqueous solubility of CAT12, a fraction of it remains partitioned in water even in the presence of liposomes, but we found that its mobility is differently affected by different compounds in the solution, thus suggesting a location at the liposome external interface (Scheme 1). In these conditions, competitive interaction of the liposome between the dendrimer and the cationic EPR probe occurs, which modulates the partitioning (relative percentage) of the probe between the external solution (free component) and the internalization of the CAT12 chain into the liposomes (interacting component).

By subtracting the computed free component in Figure 3A from the total spectrum in the same figure, the interacting component is obtained (Figure 3B). The interacting component shows the resolution of the anisotropies of the magnetic parameters. This indicates the slowing down of the motion, consequently showing the interactions of the spin probe embedded in the liposome membrane. In detail, the CAT12 chain is inserted into the membrane, while the positively charged CAT groups electrostatically interact with the phosphate groups on the liposome surface. This interacting component is simulated as shown in Figure 3B. The τ value (4 ns, in the legend of Figure 3B) is significantly higher, when compared to the free component (Figure 3A), and indicates quite strong electrostatic interactions occurring on the liposome surface.^30−32^ On the other side, the parameter ⟨*A*⟩ (15.8 G, legend of Figure 3B) indicates a lower micropolarity with respect to the free component (Figure 3A). The polarity reduction further supports the occurrence of electrostatic interactions, where the charges neutralize.

In addition to τ and ⟨*A*⟩, other parameters are useful to clarify the interaction characteristics of dendrimer–liposome systems, as follows:

(a) The total intensity (obtained by double integration of the spectra), which measures the solubility of CAT12 (mimicking lipids) in the systems (in arbitrary units = arb unit). An increased “solubility” corresponds to an increased concentration in the systems.
(b) The relative percentage of the interacting component.
(c) The order parameter *S* for the interacting component. This parameter, also obtained from computation, measures the order of lipid aggregates and changes from 0 (no order) to 1 (maximum order). In several cases, the fitting between the experimental and the computed line shape improves when both τ and *S* are included in the calculation. However, the simultaneous variations of τ and *S* significantly increase the error in the values. Therefore, it is usually preferred to change only one of the two parameters maintaining constant the other, mainly to follow the structural variations in a series of spectra from similar systems, the same as in the present case.

**Figure 4:**
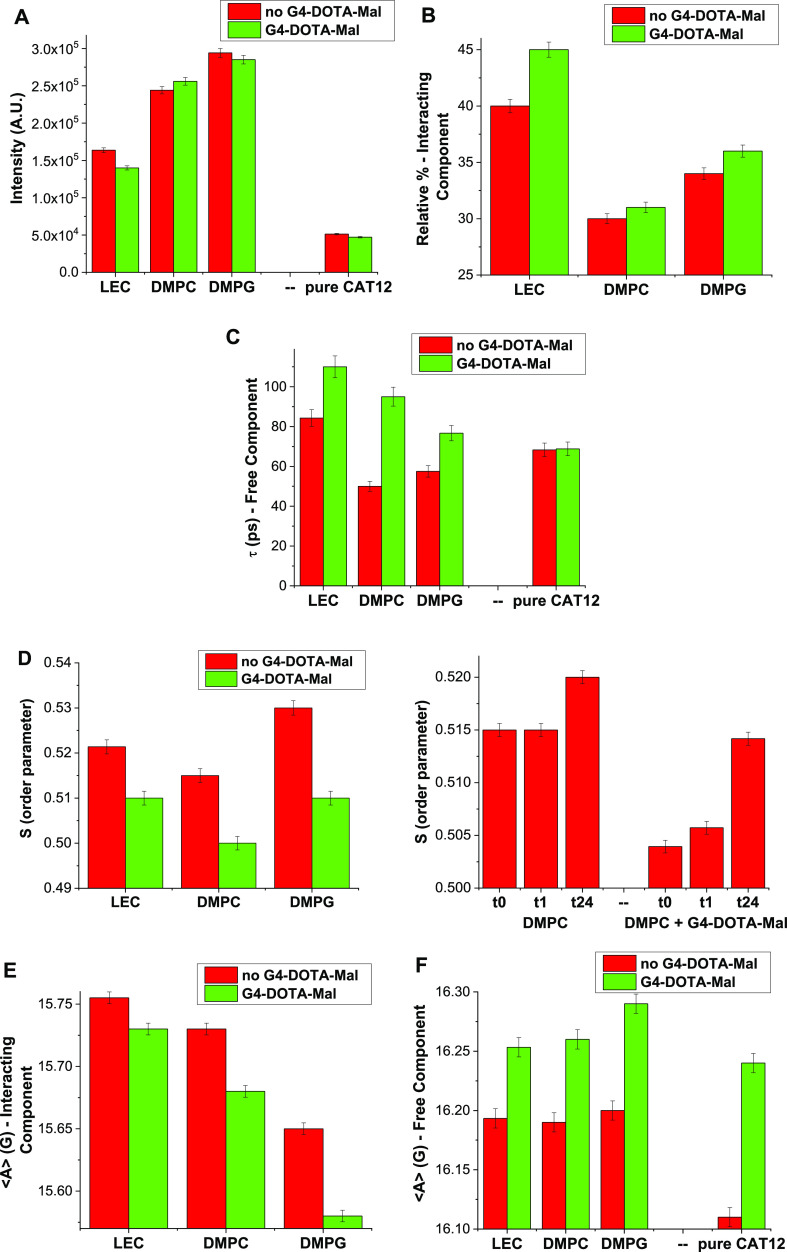
Variations of the EPR spectra intensity (A), relative % of the interacting component (B), τ of the free component (C), *S* of the interacting component (D), and ⟨*A*⟩ of the Interacting (E) and Free (F) components, for the three different liposomes in the absence and presence of G4-DOTA-Mal.

With this in mind, Figure 4 shows the variations in the spectral intensity (A), the relative % of the interacting component (B), τ of the free component (C), *S* of the interacting component for a constant τ = 5 ns value (D), and ⟨*A*⟩ of the interacting (E) and free (F) components, for CAT12 in solution with the three different liposomes, and in the absence and presence of G4-DOTA-Mal. Further comments on the results in Figure 4 are presented in Section S5.4 of the SI. The main characteristics of the various systems deduced from the data in Figure 4 are sketched in Scheme 1 and described/discussed in the following.

##### For the System “Liposome + CAT12”

In the absence of G4-DOTA-Mal, the results show that CAT12 is able to insert in the bilayer structure of liposomes (Scheme 1), where the intensity reports about an increased solubility (concentration) of CAT12 into the liposomes in the series LEC < DMPC < DMPG (Figure 4A). Here, it is interesting to note that phospholipids are used at a concentration of 25 mM. Therefore, assuming that all lipids are solubilized, the theoretical CAT12/phospholipid ratio is 1:25. However, the interacting component involves only 30–40% of the probes; therefore, the theoretical ratio becomes about 1:75. The heterogeneity of LEC ingredients may be responsible for a lower concentration of free CAT12 in LEC liposome solutions. Thus, the free component is mainly reduced since the relative percentage of the interacting component (Figure 4B) is the highest with LEC with respect to the other liposomes. This last result indicates that the surface heterogeneity of LEC liposomes favors electrostatic interactions between the positively charged CAT and phosphate groups on the LEC liposome surface. On the other side, the negative charge of the DMPG surface also enhances the electrostatic interactions with the positive CAT group. This provokes a slight increase of membrane integration (Figure 4A) and the percentage of the interacting component (Figure 4B) for DMPG when compared to DMPC. The enhanced interactions (insertion into the bilayer and electrostatic interaction on the liposome interface) of CAT12 embedded into DMPG liposomes are finally proved by the higher-order parameter (Figure 4D) and the lower polarity (measured by ⟨*A*⟩ in Figure 4E) when compared to the other liposomes.

##### For the Systems “Liposome + G4-DOTA-Mal + CAT12”

The addition of G4-DOTA-Mal to the liposomes provides the following effects: (i) an increase in the percentage of the interacting component (Figure 4B), (ii) an increase of τ (measuring the interaction strength, Figure 4C) and ⟨*A*⟩ (measuring the polarity, Figure 4F) for the free component, and (iii) a decrease of the order parameter *S* (Figure 4D) and ⟨*A*⟩ (Figure 4E) for the interacting component. The increase in the percentage of the interacting component is unexpected since the presence of a positively charged dendrimer, which prefers to bind to the liposome surface, should impede the insertion of CAT12, which is also positively charged. However, CAT12 needs to escape from the solution due to both electrostatic repulsion with the dendrimer approaching the liposome surface and increased instability of the hydrophobic chain caused by increased ionic strength in the solution. This provokes increased CAT12 solubilization in the liposomes. The variations of the parameters in Figure 4 are indeed quite small but support the occurrence of weak dendrimer–liposomes interactions (Scheme 1). These interactions slightly perturb the liposome structure, as tested by a decrease in the lipid order (order parameter) and an increase in the polarity (⟨*A*⟩) of the interacting component. These variations are larger for DMPG and smaller for LEC. Conversely, the increase in the percentage of the slow component is larger for LEC. This is accompanied by a decrease in the spectral intensity for LEC (Figure 4A), while this behavior is not found for the other liposomes. Therefore, G4-DOTA-Mal interactions with LEC perturb the solubilization (=membrane integration) of free CAT12 probes in LEC liposome solutions, thus decreasing the intensity and increasing the percentage of interacting probes. As shown in Scheme 1, the EPR results indicate a stronger structural perturbation tailored by cationic G4-DOTA-Mal on anionic DMPG liposomes. Contrarily, more stable dendrimer–liposome adducts are formed by LEC. The postulated surface interactions between G4-DOTA-Mal and LEC are probably related to the heterogeneity of lecithin ingredients on the liposome interface, which enhances surface interactions. Indeed, lecithin contains the zwitterionic PC and PE lipids: more unsaturated lipids will result in packing defects that facilitate dendrimer attachment and/or adsorption at the interface, where the phosphate group is located, without perturbing the lipid order. The interactions of cationic CAT12 at the interface between G4-DOTA-Mal and liposomes (Scheme 1) is also measured by an increase of τ (Figure 4C) and ⟨*A*⟩ (Figure 4F) for the free component in the liposome solution from the absence to the presence of G4-DOTA-Mal. Surface interactions of G4-DOTA-Mal with LEC are additionally proven by fluorescence anisotropy experiments (further details in Figure S9, SI). The results in Figure S12 using the TMA-DPH fluorescent probe clearly indicate a more decisive hydrophilic interaction with the dendrimer for LEC. Using DPH, the lower starting r/r0 values for LEC indicate a more fluidic membrane with respect to DMPC and DMPG. DMPG shows a progressive negative slope, which may be related to an increased fluidity at higher dendrimer concentrations.

Both fluorescence and EPR results show that DMPC possesses a more compact bilayer structure when compared to the other liposomes. Thus, DMPC outlines the lowest interactions at the interface.

The EPR results are in agreement with ZP data (Table S2), thus confirming the compensation of the negative surface charge in the cases of LEC and DMPG liposomes for turning them to nearly neutral surface charge. Conversely, nearly neutral DMPC liposomes do not undergo charge neutralization through the addition of cationic G4-DOTA-Mal (Table S2). Indeed, electrostatic interactions between anionic DMPG and LEC and cationic G4-DOTA-Mal are expected. Therefore, without a doubt, electrostatic interactions are the driving forces between liposomes and dendrimer surfaces. Moreover, the interactions between the DMPC liposomes and G4-DOTA-Mal dendrimers are mainly driven by dipole–ionic bonds and hydrogen bonds. The multiple H-bond interactions of G4-DOTA-Mal on the neutral surface can cause perturbations of the membrane fluidity,^50−52^ which also happen in the case of LEC and DMPC. With respect to EPR results, H-bonds are less significant for DMPC, probably related to the neat and compact structure. In conclusion, the radical surfactant, CAT12, mimicking the phospholipid behavior, reveals to be a good probe to characterize the interactions between liposomes and G4-DOTA-Mal. This probe distributes differently in different liposome solutions in the absence and presence of G4-DOTA-Mal, thus monitoring the liposome–dendrimer interactions.

#### Analysis of the EPR Spectra of G4-DOTA-Mal–Cu(II) Complexes Interacting with Liposomes

The complexation of G4-DOTA-Mal (1.56 mM, corresponding to 9.36 mM in DOTA groups) with Cu(II) ions is analyzed at three different concentrations of Cu(II), 7, 14, and 28 mM (corresponding to 0.75, 1.5, and 3 molar ratios between Cu(II) and DOTA in the absence and presence of three different liposomes (LEC, DMPC, and DMPG)) and at different equilibration times up to 24 h. Longer equilibration times (up to 15 days) showed poor spectral variations, indicating the stability of the liposomes solutions, despite the high phospholipid concentration. Liposome solutions are added to the solutions of preformed glycodendrimer–Cu(II) complexes. For comparison, also, the binary Cu(II)–liposomes and Cu(II)–G4-DOTA-Mal solutions are analyzed by EPR.

The computation of Cu(II) spectra is performed by the same procedure used for computing the EPR spectra of the nitroxide radicals,^41^ but in this case, the parameters and information extracted from computations are different. First, the *A_ii_* components of the **A** tensor for the coupling between the electron spin and the nuclear spin (Cu, *I* = 3/2) characterize the type and geometry of the Cu(II) complex, together with the *g_ii_* components of the **g** tensor for the coupling between the electron spin and the magnetic field. The attribution to a certain Cu(II) coordination and geometry is based on the comparison with the *A_ii_* and *g*_*ii*_ values reported in the literature for similar systems.^32−42^ This computation procedure has the advantage to also provide correlation time for motion, τ, which measures the mobility of the Cu(II) complex.

Samples “Cu(II)–liposomes” outlined an abundant “free component” for the Cu(II) coordination with four oxygen sites in a square planar geometry and fast mobility, exemplified for LEC in Figure S11 in the SI, bottom spectrum. This free Cu–O4 component is most probably characterized through the coordination of Cu(II) with four water molecules. On the contrary, only the Cu(II)–LEC sample, due to the heterogeneity of the lecithin components, additionally depicts the presence of the so-called “weakly interacting” component (Figure S11 in the SI, bottom spectrum); on the basis of the partial resolution of the **g** and **A** anisotropies, this component is attributed to ions weakly interacting with the LEC surface in slow mobility conditions (trapped at the interface). The heterogeneity of the LEC interface due to the PE and PC lipids well accounts for this behavior.

The intensity of the weakly interacting component in the case of LEC–Cu(II) system decreases, relative to the free component, as the Cu(II) concentration increases from 7 mM to 14 and 28 mM due to saturation of the weakly interacting sites on the surface of LEC liposomes. Therefore, only the Cu(II)–O4 coordination is visible at the highest Cu(II) concentration for LEC liposomes and at all concentrations for DMPC and DMPG liposomes.

**Figure 5:**
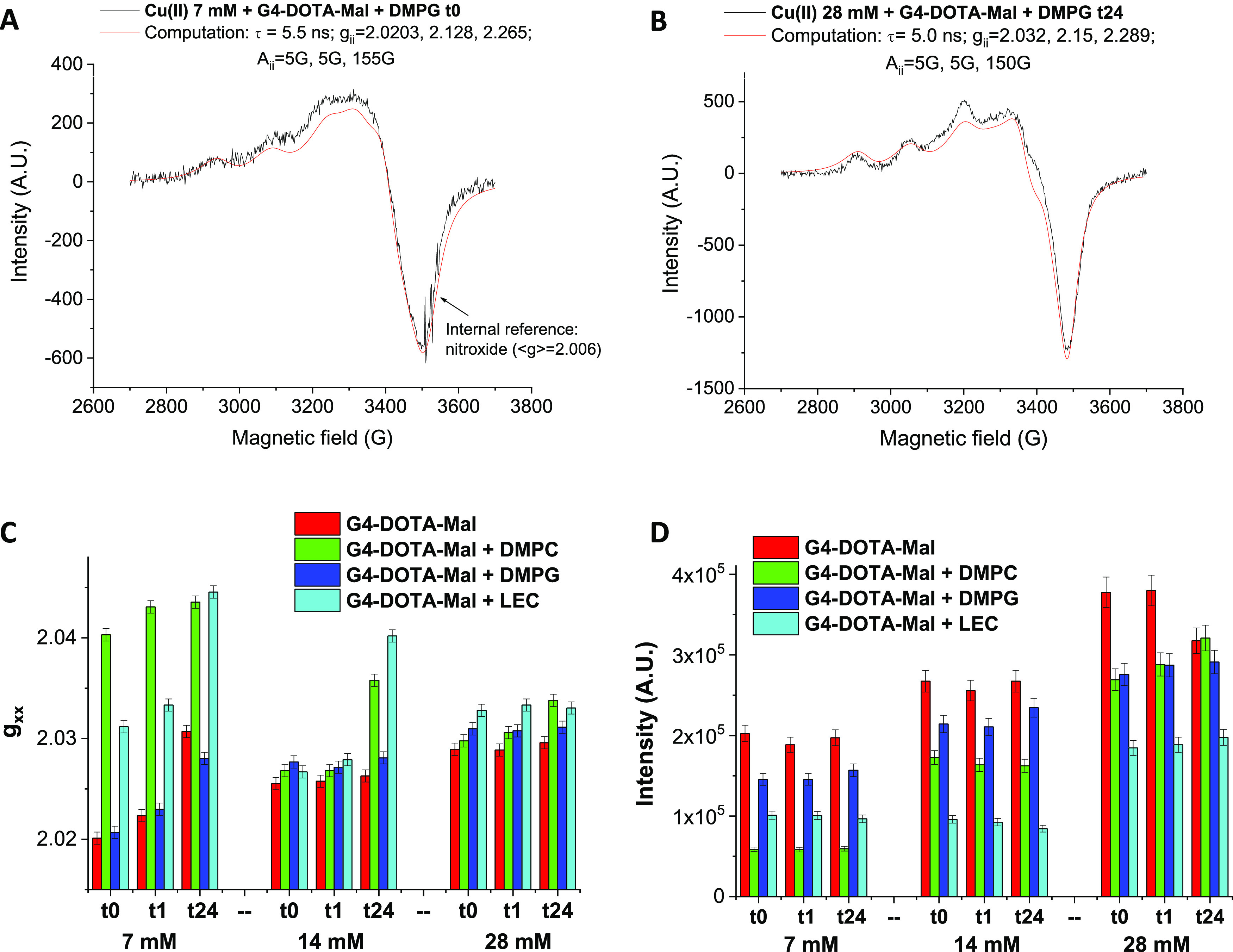
Experimental and computed EPR spectra of G4-DOTA-Mal–Cu(II) 7 mM + DMPG at *t*_0_ (A) and G4-DOTA-Mal–Cu(II) 28 mM + DMPG at *t*_24_ (B) and *g_xx_* values (C) and intensity values (D) for the various systems.

**Scheme 2:**
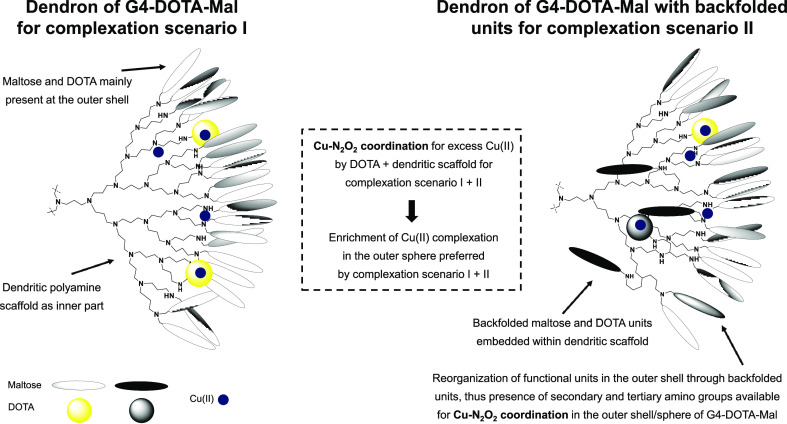
Complexation of Cu(II) by G4-DOTA-Mal and Molecular Rearrangement of G4-DOTA-Mal by Backfolded Functional Groups Core–shell model suggested for G4-DOTA-Mal (left).^52^ Backfolded maltose units in G4-DOTA-Mal, inducing the presence of amino groups for Cu(II) coordination in the outer sphere. No core–shell structure, resembling the “fluffy model” of the open-shell architecture of maltose-modified hyperbranched poly(ethyleneimine) (right).^53^ Part structure (dendron) of G4-DOTA-Mal for exemplifying Cu(II) coordination. Excess Cu(II) may only be complexed by amino groups of the G4-DOTA-Mal scaffold, as shown in scenarios I and II.

The other binary system, constituted by Cu(II) and G4-DOTA-Mal, shows completely different spectra when compared to the Cu(II)–liposome binary systems: the spectra of Cu(II)–G4-DOTA-Mal at all concentrations (7–28 mM), both in the absence and presence of the liposomes, are only constituted by the so-called interacting component, as shown in the examples in Figure 5A,B. The interacting behavior is accounted for by the τ value (5.5 and 5 ns; Figure 5A,B, respectively), obtained from the computations (red lines in Figure 5A,B). These τ values indicate a slow-moving complex due to Cu(II) ions binding within the G4-DOTA-Mal scaffold. The magnetic parameters *g_ii_* and *A_ii_* (also reported in the legends of Figure 5A,B) indicate a square planar Cu–N_2_O_2_ coordination but with strong orthorhombic distortion. This distortion is caused by stronger coordination with two nitrogen sites, probably those present in the DOTA group. The two oxygen sites, which coordinate the ions, are probably water molecules. Alternatively, it is also reasonable to think that two nitrogen atoms of the dendritic scaffold of G4-DOTA-Mal may be involved in Cu–N_2_O_2_ coordination, especially at the highest Cu(II) concentration (28 mM), as shown in Scheme 2. Indeed, DOTA ligands are saturated by Cu(II) ions, when considering the Cu(II) complexation by G4-DOTA-Mal at the highest DOTA/Cu(II) molar ratio (1:3).

The use of an internal reference system (a nitroxide radical with ⟨*g*⟩ = (*g_xx_* + *g_yy_* + *g_zz_*)/3 = 2.006, indicated with an arrow in Figure 5A) allows an accurate measurement of the *g_ii_* values (error in the third decimal). In this Cu(II)–dendrimer binary case, the line shape modifies by increasing Cu(II) concentration. This modification in the line shapes is described and discussed together with those obtained for the ternary liposome–Cu(II)–dendrimer systems in the following.

Indeed, liposome solutions (LEC, DMPC, and DMPG) are added to the Cu(II)/G4-DOTA-complex solution to clarify the complex interaction characteristics between the three components on the basis of the mobility parameter, τ, and the magnetic parameters, *g_ii_* and *A_ii_* (identifying Cu(II) complex coordination), obtained from the computation process.

Figure 5B shows an example of experimental (black line) EPR spectrum (in red the computation) obtained after the addition of the liposome DMPG to Cu(II)/G4-DOTA-Mal complex solution, recorded at an equilibration time of 24 h and at a Cu(II) concentration of 28 mM. The spectrum in Figure 5B is constituted by a single interacting component, similarly to the spectrum in the absence of the liposomes in Figure 5A. However, the main parameters used for computations and listed in the legends of Figure 5A,B differ in the two computations. We found that *g_xx_* and spectral intensity parameters (Figure 5C,D, respectively) are the most informative to describe the structural variations of the Cu/G4-DOTA-Mal complex from the absence to the presence of different liposomes at different equilibration times and Cu(II) concentrations.

**Scheme 3:**
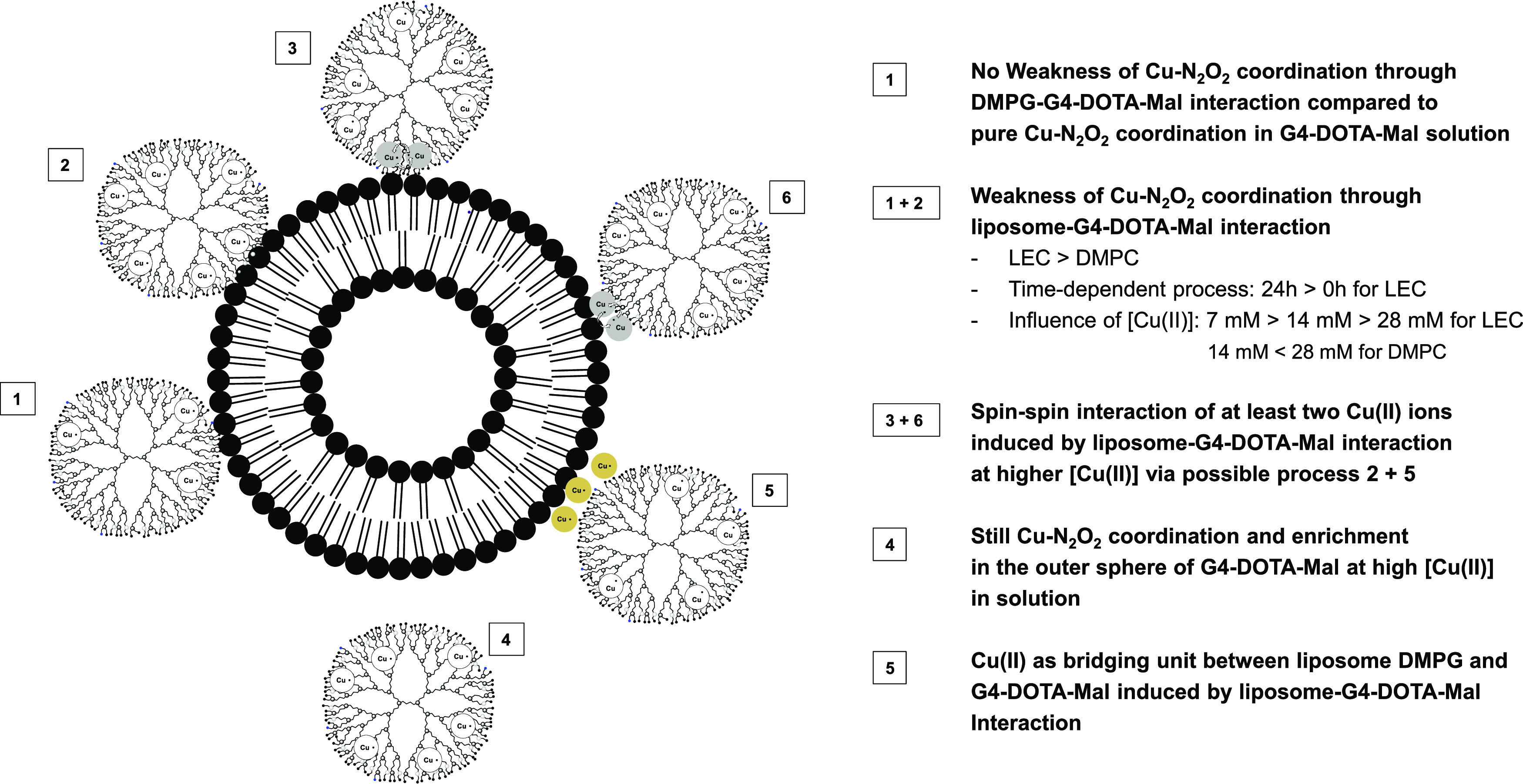
Different Complexation Behaviors of Cu(II) with the Dendrimer in the Absence and Presence of the Liposomes

To analyze the *g_xx_* data, it must be taken into account that an increase of *g_xx_* corresponds to a weaker interaction and/or a lower number of nitrogen sites coordinated to Cu(II). On the other side, a decrease in intensity (Figure 5D) may arise from a decrease of ion concentration in the systems and/or strong spin–spin interactions, which provoke a significant line broadening. Consequently to this broadening, the corresponding EPR signal almost “disappears” in the magnetic field range of analysis. Thus, the following Cu(II)/dendrimer interaction characteristics with the liposomes can be deduced and are sketched in Scheme 3.

First, as depicted in Scheme 2, there is a higher probability of backfolded maltose and DOTA inside of the dendritic scaffold of G4-DOTA-Mal, also described as a lower probability to form a dense-shell PPI glycodendrimer.^52^ Thus, with this molecular rearrangement of maltose and DOTA units, the nitrogen atoms of the dendritic scaffold are available on the outer surface of G4-DOTA-Mal, ready to complex the excess Cu(II) with a Cu–N_2_O_2_ coordination. Potential oxygen ligands can be water molecules and/or oxygen-containing surface groups of liposomes (complexation scenario II in Scheme 2). On the other hand, the Cu–N_2_O_2_ coordination of the excess Cu(II) may also occur in the inner and outer spheres of the dendritic scaffold of G4-DOTA-Mal besides Cu(II) complexation by the DOTA ligand (complexation scenario I in Scheme 2).

The first consideration (Scheme 3, cases 1 + 2) is that the addition of LEC and DMPC liposomes to Cu(II)/G4-DOTA-Mal complex decreases the interaction strength (increase of *g_xx_* in Figure 5C) between Cu(II) and the nitrogen ligands of the dendrimer. This mainly happens at the lowest Cu(II) concentration and is necessarily ascribed to the interactions between the Cu(II)–dendrimer complexes and liposomes. This perturbation effect is almost immediate (at *t*_0_ and *t* = 1 h) for DMPC, while it needs time (up to 24 h) for LEC. Conversely, in the presence of DMPG, the interaction strength between Cu(II) and the nitrogen ligands remains almost unchanged with respect to the complex in the absence of liposomes (Scheme 3, case 1). In this case, Cu(II) ions, maintaining the Cu–N_2_O_2_ coordination, are probably directly interacting with the negative groups (phosphate) at the DMPG interface (the two oxygen ligands) and probably work as a bridge between the liposomes and the two nitrogen sites of the dendrimers (Scheme 3, cases 4 + 5). Here, it is postulated the complexation scenario II (Scheme 2).

The interaction strength measured by *g_xx_* (Figure 5C) decreases for all systems by increasing the Cu(II) concentration (from 7 mM to 14 and 28 mM) and the equilibration time (from 0 to 24 h). Thus, Cu–N_2_O_2_ coordination in Cu(II)–G4-DOTA-Mal complexes is weakened both over time and by increasing Cu(II) concentration from 7 to 14 mM, both in the absence and in presence of all liposomes. The increase in Cu(II) ions at the dendrimer or dendrimer/liposome interface provokes charge repulsions, which are also disturbing the Cu(II)–N bonds. At the highest Cu(II) concentration (28 mM), the complex-weakening effect played by the various liposomes becomes quite small. The excess ions localize in the Cu–N_2_O_2_ coordination more internally to the dendrimer (scenario II, Scheme 2), and, consequently, the perturbations due to liposome–dendrimer interactions are less effective.

Moreover, the increase in the *g_xx_* parameter by adding LEC and DMPC to the Cu(II)–dendrimer complex at the Cu(II) concentration of 7 mM (Figure 5C) is accompanied by a decrease in intensity (Figure 5D). The decrease in intensity is mainly ascribable to spin–spin interactions occurring between Cu(II) ions, which concentrate at the dendrimer–liposome interface (Scheme 3, cases 3 and 5). Again, both complexation variants I and II (Scheme 2) can be involved to initiate such spin–spin interactions due to next-to-next complexation locations within the dendritic scaffold of G4-DOTA-Mal at the dendrimer–liposome interface but not really between two G4-DOTA-Mal macromolecules on the liposome surface.

It is worth noting that the intensity increases with an increase in the concentration of Cu(II) but far from a logic of proportionality. This further demonstrates that the Cu(II) ions concentrate (spin–spin interactions) at the G4-DOTA-Mal surface/interface, both in the absence and presence of liposomes. The Cu–N_2_O_2_ coordination with a weakening of the Cu(II)–N bonds is the only one visible in the EPR spectra at all Cu(II) concentrations, supporting the hypothesis of Cu(II) coordinating nitrogen sites into the dendrimer scaffold and concentrating at the interface. Such Cu(II) enrichment in the outer sphere of dendrimers is already described in a previous study on similar systems using dense-shell PPI glycodendrimers.^51^ The lowest variation in intensity by increasing the Cu(II) concentration is found in the presence of LEC. In this case, the heterogeneous liposome surface favors the aggregation of ion/dendrimer complexes on the LEC surface itself.

All of these considerations show that the EPR analysis provides useful interaction properties of G4-DOTA-Mal with respect to its ability to selectively complex Cu(II) ions and then interact with the external liposome surface. These interactions are modulated by the type of liposome, the copper concentration, and the equilibration time.

## Conclusions

The aim of this study is to deeply characterize the Cu(II)-complexing ability of a DOTA-functionalized fourth-generation PPI glycodendrimer with a dense maltose shell (G4-DOTA-Mal) and its surface/interface interactions with various liposomes (LEC, DMPC, and DMPC/DMPG 3%, termed as DMPG), without being internalized or destroying/disrupting the liposome membrane structure. For such a basic study, it is preferable to use stable liposomes instead of heterogeneous and poorly stable cell cultures. Surface-driven interactions of potential polymeric therapeutics are desirable to develop supramolecular Cu(II)-complexing drugs for treating various pathologies where Cu(II) has revealed a harmful role.

Therefore, this multitechnique study analyzes liposomes in the absence and presence of G4-DOTA-Mal and in the absence and presence of different molar ratios of Cu(II). The combination of DLS, ZP, and cryo-TEM shows that unilamellar, long-term stable, and nonaggregating liposomes are obtained, whose structure characteristics are retained also in the presence of G4-DOTA-Mal. The dendrimers in the absence of liposomes form aggregates, which breakdown when dendrimer–liposome interactions occur.

EPR, supported by fluorescence anisotropy, helps us to clarify the interaction characteristics of G4-DOTA-Mal in the absence and presence of Cu(II) combined with the presence of the liposomes.

The use of the specific spin probe CAT12 clarifies that noncovalent surface-driven interactions (electrostatic interactions, H-bonds, ion dipoles) of G4-DOTA-Mal with all liposomes are present, proving the presence of different interaction adducts (Scheme 1). Especially, ionic interactions mainly occur between cationic G4-DOTA-Mal and the anionic DMPG surface. Weak interactions arise between G4-DOTA-Mal with nearly neutral DMPC liposomes, attributed to a compact liposome structure. This liposome surface poorly adapts to the dendrimer surface. Conversely, competitive interactions are evident at the anionic LEC interface, assuming ionic and H-bond interactions with G4-DOTA-Mal. This is mainly ascribed to the heterogeneous LEC surface. The mixture of saturated and unsaturated alkyl chains of the two zwitterionic PC and PE lipids, characterized by a smaller ethanolamine group with the potential for H-bonds and a larger choline group, creates structural defects which favor the dendrimer–liposome interactions at the LEC interface. Therefore, stable adducts are formed between LEC and G4-DOTA-Mal, but the weak interactions favor fast exchange with the bulk solution.

The EPR spectra of the Cu(II)/G4-DOTA-Mal solutions indicate that cationic G4-DOTA-Mal is an excellent copper complexing agent. A Cu–N_2_O_2_ coordination with an orthorhombic distortion of a square planar geometry takes place by coordinating two nitrogen sites of the DOTA group and two water molecules. However, we cannot exclude that also dendrimer-internal nitrogen sites and the phosphate ions of PBS buffer are involved in the coordination.

By adding DMPC and LEC liposomes to Cu(II)/G4-DOTA-Mal complexes (Cu–N_2_O_2_ coordination), a weakening of the Cu(II)–nitrogen ligands’ binding strength occurs due to competitive liposome–dendrimer and Cu(II)–dendrimer binding and is influenced by the liposome characteristics (*e.g*., composition, surface charge, and surface heterogeneity) and the dendrimer concentration. The weakening of Cu–N_2_O_2_ coordination is still available at the lowest Cu(II) concentration in the presence of DMPC since the earliest incubation times (Scheme 3). At later incubation times and higher dendrimer concentrations, LEC liposomes become more perturbative of the Cu(II)–dendrimer complexes due to LEC–dendrimer interactions. Fluorescence anisotropy results, performed using a lipid concentration of 500 μM and increased dendrimer concentration (from 0 to 100 μM), also supported the finding of LEC–dendrimer interactions. In Figure S12, the maximum interaction with the TMA-DPH probe was found at the same dendrimer/lipid molar ratio used for EPR. A different interacting mode is hypothesized for DMPG, for which perturbation of the complex stability is smaller. We hypothesize that this negatively charged liposome interacts with the whole complex at the interface without weakening the postulated Cu–N_2_O_2_ coordination (Scheme 3).

At high Cu(II) concentrations, spin–spin interactions between close ions prevail at the dendrimer/liposome interface, mainly in the cases of LEC and DMPC (Scheme 3). In summary, G4-DOTA-Mal PPI glycodendrimer forms stable Cu(II) complexes and outlines the desired surface-driven interactions with the membrane surfaces of the different tested liposomes. The binding strength of G4-DOTA-Mal toward liposome systems is tailored by the membrane composition, Cu(II) concentration, and interaction time.

Moreover, the results suggest that G4-DOTA-Mal and its Cu(II) complexes do not undergo internalization by any liposome and that G4-DOTA-Mal is usable as a Cu(II) complexation agent in the presence of bilayer structures, needed for future *in vitro* study. This is smoothly attributed to dynamic and overtime stable glycodendrimer–liposome interactions without destroying the bilayer. Further studies are in progress to deepen the cell membrane interaction of G4-DOTA-Mal with more sensitive labels and the understanding of the biological action of G4-DOTA-Mal for capturing Cu(II).

## Supporting Information Available

Additional experimental details (materials, devices, synthesis of G4-DOTA-Mal, experimental procedures) and data on DLS (also dealing on the stability of liposomes obtained by means of the extrusion method), ZP and fluorescence anisotropy experiments: comments and associated figures (Figures S1–S12) and tables (Tables S1 and S2), and further comments on Figure 4 about the intensity of the EPR spectra (PDF)
