# Supplementary material for: DOTA Glycodendrimers as Cu(II) Complexing Agents and Their Dynamic Interaction Characteristics toward Liposomes
Source: Langmuir. 2020 Sep 30;36(43):12816–29. doi: 10.1021/acs.langmuir.0c01776 (PMC8015221; doi:10.1021/acs.langmuir.0c01776)
Supplement: Supplementary file 1 — la0c01776_si_001.pdf [file la0c01776_si_001.pdf]

Electronic Supporting Information (SI)

DOTA-Glycodendrimers as Cu(II) complexing agents and their dynamic interaction characteristics towards liposomes

Marianna Carone[1], Silvia Moreno[2], Michela Cangiotti[3], Maria Francesca Ottaviani[3], Peng Wang[2], Riccardo Carloni[*3], Dietmar Appelhans[*2]

[1]Department of chemistry and biochemistry, University of Bern, Bern, Switzerland

[2]Leibniz Institute for Polymer Research Dresden, Hohe Strasse 6, D-01069 Dresden, Germany

[3]Department of Pure and Applied Sciences, Università degli studi di Urbino "Carlo Bo", Urbino, Italy

* Corresponding authors: applhans@ipfdd.de; riccardocarloni93@gmail.com

Number of pages: 21

Number of figures: 12

Number of schemes:0

Number of tables: 2

Table of Contents:

Experimental details:

materials; devices; details on the synthesis of the dendrimer; experimental procedures.

Additional information for the Results and Discussion:

DLS and ZP study providing size and charge of dendrimers and/or liposomes in the absence and presence of Cu(II); long-term stability of liposomes at different temperatures by DLS; further examples of CAT12 EPR spectra, details about Figure 4 in the manuscript; fluorescence anisotropy study.

1. Materials

PPI dendrimers of DAB-Am64 were supplied by SyMO-Chem (Eindhoven, Netherland). D(+)-Maltose monohydrate, sodium borate, borane–pyridine complex (BH_3*Py, 8 M solution in THF) and anhydrous dimethylsulfoxide were used as purchased from Fluka (Darmstadt, Germany). 1,4,7,10-Tetraazacyclododecane-1,4,7,10-tetraacetic acid mono(N-hydroxysuccinimide ester) (DOTA-NHS-ester) was purchased from Macrocyclis (Dallas, TX). Nomenclature of PPI dendrimers was from the suggestion of the literature [Tomalia, D. A.; Rookmaker, M. In Polymer Data Handbook, 2nd ed.; Mark, J. E., Eds.; Oxford University Press: Oxford, New York, **2009**, 979-982]. Regenerated cellulose dialysis membranes with a molecular weight cut-off (M_{WCO}) of 1000 and 2000 were acquired from Fisher (Pittsburgh, PA).

Phospholipids:

Egg lecithin:

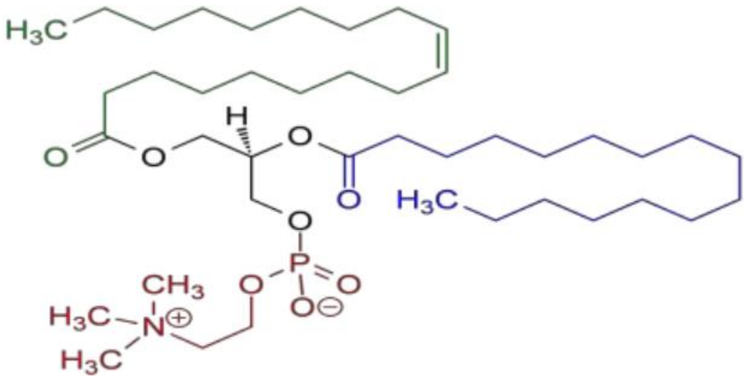

Dimyristoylphosphatidylcholine (DMPC):

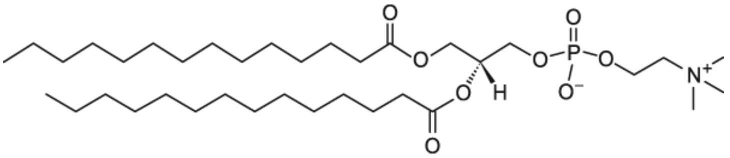

1,2-Dimyristoyl-sn-glycero-3-phospho-rac-(1-glycerol) sodium salt (DMPG)

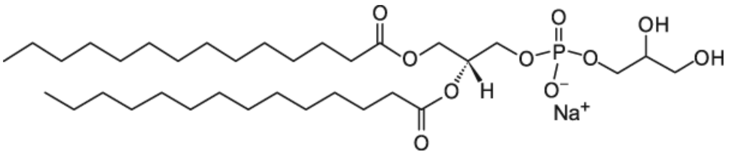

2. Devices

NMR measurements were carried out on a Bruker DRX 500 NMR spectrometer operating at 500.13 MHz for ^{1}H NMR using D_2O or DMSO-d_6 as a solvent. Sodium 3-(trimethylsilyl)-3,3,2,2-tetradeuteropropionate was added for internal calibration (δ (^{1}H) = 0 ppm). The signal assignments were performed by a combination of 1D and 2D NMR experiments using the standard pulse sequences provided by Bruker.

Time of Flight Mass Spectrometry (MALDI-TOF MS) investigations were performed on a Bruker Autoflex Speed TOF/TOF in reflector or linear modes, respectively, and positive polarity by pulsed smart beam laser (modified Nd:YAG laser). The ion acceleration voltage was set to 20 k. For the sample preparation, the substances were mixed with 2,5-dihydroxy benzoic acid as matrix, both dissolved in millipore water.

Zeta-potential (ZP) and dynamic light scattering (DLS) measurements were performed using a Malvern Zetasizer Nano ZS system (model ZEN3600, Worcestershire, UK) equipped with a standard 633 nm laser. Perkin Elmer Optima 7000 DV with a spectral range of 160-900 nm and a resolution < 9 pm were used for carrying out ICP-OES. 2% nitric acid solution was used as solvent.

Cryo-TEM images were acquired using Libra 120 microscope (Carl Zeiss Microscopy GmbH, Oberkochen, Germany) at an acceleration voltage of 120 kV.

EPR experiments were performed by using an EMX-Bruker spectrometer working at X-band (9.5 GHz).

3. Synthesis of DOTA- and maltose modified 4th generation poly(propyleneimine)dendrimer (G4-DOTA-Mal)

DOTA-modified 4th generation poly(propyleneimine)dendrimer (G4-DOTA). DAB-Am64 (0.1 g, 0.0139 mmol) and DOTA-NHS-ester (0.124 g, 0.125 mmol) were reacted in anhydrous DMSO (20 mL) under a N_2 atmosphere. The reaction mixture was stirred overnight followed by dialysis against water (6 times, 2 L) for 2 days using a dialysis membrane with an MWCO of 1000. After freeze drying, a viscous liquid of the product G4-DOTA was quantitatively obtained. ^{1}H NMR spectrum is presented in Figure S1.

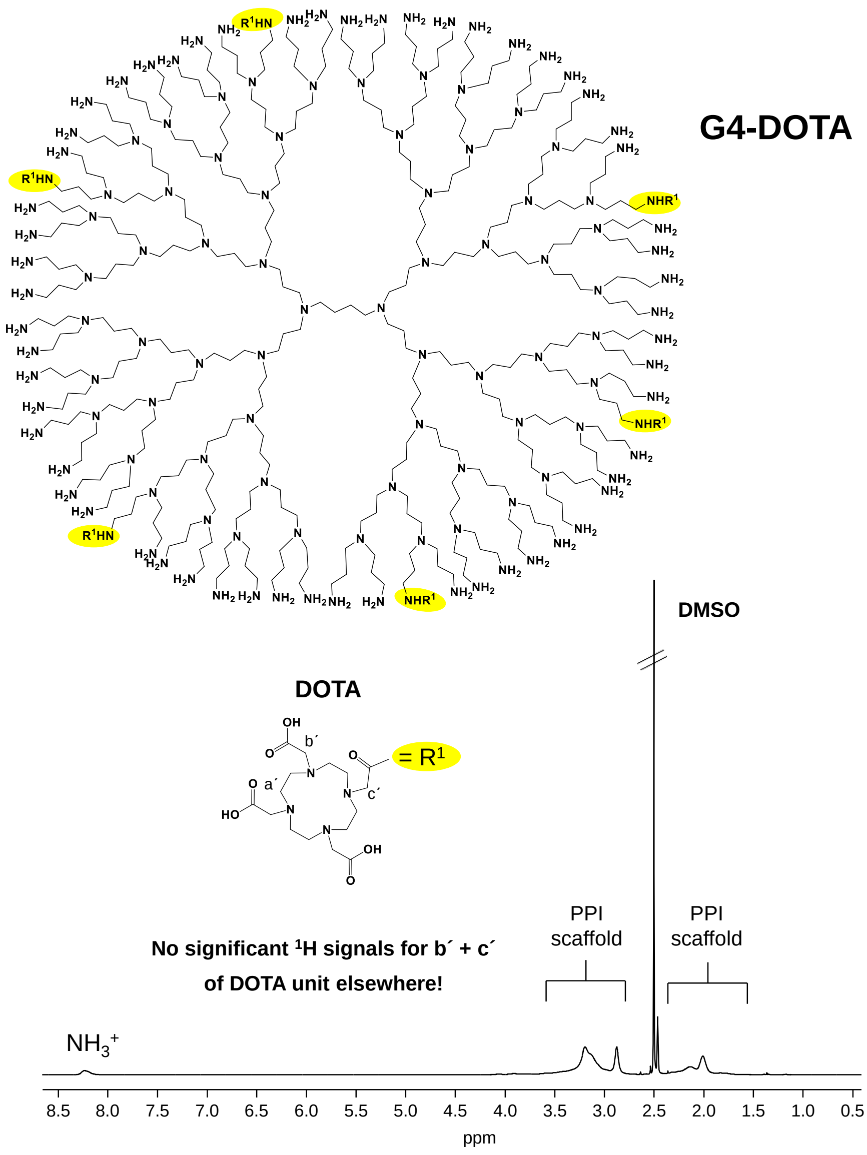

Figure S1. ^{1}H NMR spectrum of DOTA-modified 4th generation poly(propyleneimine) (PPI) dendrimer (G4-DOTA) in DMSO-d$_6$. ^{1}H NMR spectrum of G4-DOTA is similar to previously published ^{1}H NMR spectrum of DOTA-modified 4th generation poly(propyleneimine) dendrimer [Xiong Z. et al., *Biomaterials Science* **2016**, 4, 1622-1629].

DOTA- and maltose modified 4th generation poly(propyleneimine)dendrimer (G4-DOTA-Mal). The G4-DOTA dendrimer (0.10 g, 0.014 mmol), maltose monohydrate (14.74 g, 41 mmol), and borane–pyridine complex (5 mL, 40 mmol, 8 M solution) were mixed in a sodium borate buffer (30 mL, 0.1 M). The reaction solution was stirred at 50 °C for 7 days, thereafter the crude product was purified by dialysis against water (6 times, 2 L) for 4 days. Further lyophilization gave rise to the formation of the G4-DOTA-Mal dendrimer with a yield of 0.400 g (95%). ^{1}H NMR signals of the DOTA unit attached on the G4-DOTA-Mal

are suppressed by the dendritic PPI scaffold and maltose units. All other typical ^{1}H NMR signals of the G4-DOTA-Mal glycodendrimer with dense maltose shell are the same as previously published [Xiong Z. et al., *Biomaterials Science* **2016**, 4, 1622-1629]: ^{1}H NMR (D_2O): δ = 1.4–2.3 (a, d, g, j, m and p); 2.3–3.35 (1′, b, c, e, f, h, i, k, l, n, o and q); 3.35–4.55 (2–6 and 2′-6′); 4.95–5.35 ppm (1). In Figure S2, ^{1}H NMR signals are only determinable for PPI-scaffold and maltose units in G4-DOTA-Mal, while degree of DOTA units attached to PPI scaffold was determined by ICP-OES. The degree of chemically coupled DOTA units on G4-DOTA-Mal was determined by inductively coupled plasma-optical emission spectroscopy (ICP-OES) through complexation of Gd(III) ions. About 6 DOTA are attached to the scaffold of dendritic poly(propyleneimine). Molecular weight of G4-DOTA-Mal is 37,500 g/mol determined by MALDI-TOF MS. Thus about 86 maltose units are attached on the dendritic PPI scaffold (Figure S2).

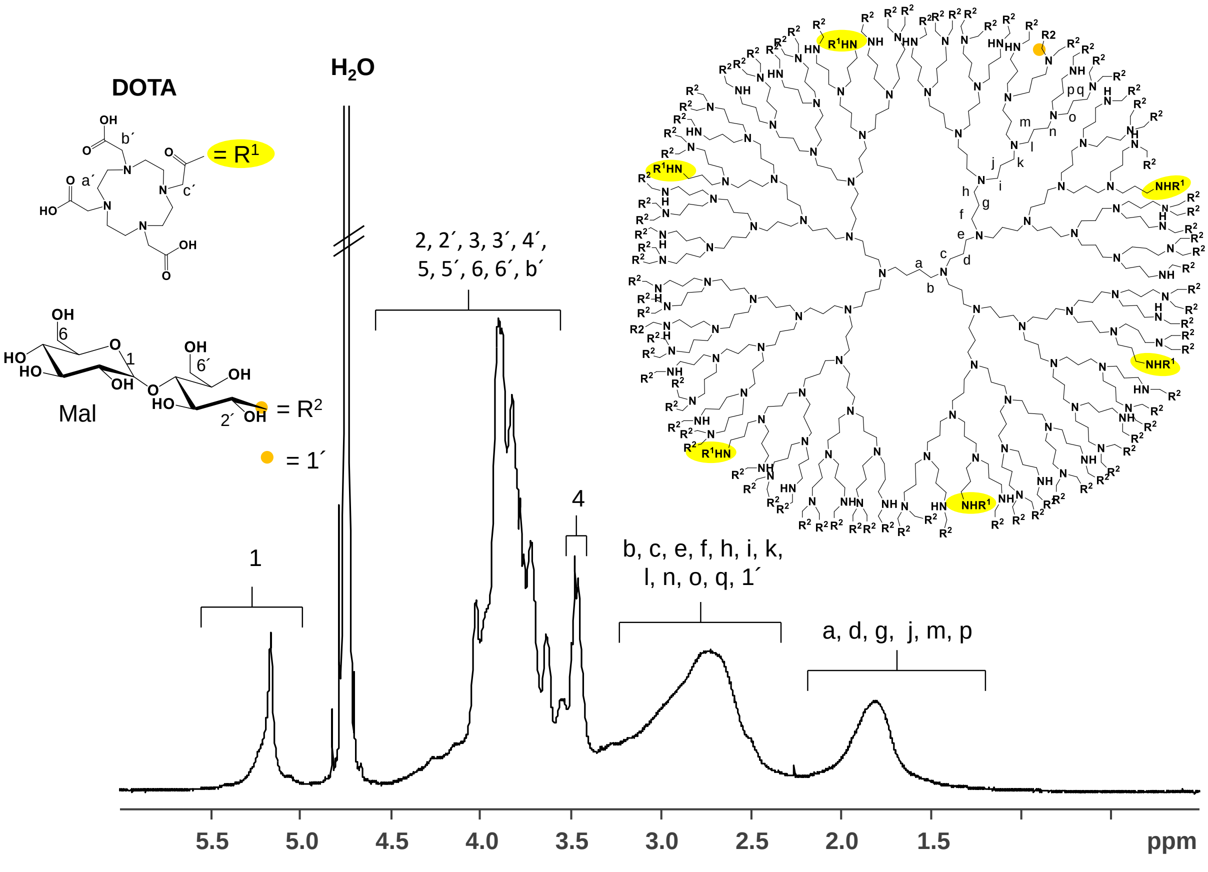

Figure S2. ^{1}H NMR spectrum of DOTA-modified 4th generation poly(propyleneimine) dendrimer with dense maltose shell (G4-DOTA-Mal) in D_2O.

4. Experimental procedures

4.1 DLS and ZP experiments

The size of particles and the polydispersity index (PDI) were measured using the DLS technique. The equilibration time was 60 sec., while the measurement angle was 173° and the number of measurements was 3 per 25 runs (run duration 5 sec). The peak size gives the z-average. All the experiments were performed at 25°C, and at different equilibration times, t = 0, 1h, and 24 h.

The surface charge was determined by using the Zetasizer Nano-ZS with a combination of electrophoresis and laser Doppler velocimetry. Electrophoretic mobility of particles was measured by using capillary plastic cells with a copper electrode, covered with gold, in order to apply an electric field.

The data evaluation was carried out by using Malvern Software from the Helmholtz–Smoluchowski equation.

First, liposomes (in the absence of dendrimers) were tested to monitor their thermal stability over time; each liposome (LEC, DMPC and DMPG) solution were divided into two aliquots and incubated at 4°C and 37°C, respectively. The liposome stability was performed after 3, 10, 20 and 30 days since the day of preparation, and at 25°C, 30°C, 37°C, and 40°C. Liposome solutions were prepared in 10 mM PBS at a concentration of 0.50 mg/mL for LEC, and 0.30 mg/mL for DMPC and DMPG.

To study the interactions of G4-DOTA-Mal - Cu(II) complex with the liposomes, the compounds were added following a specific order: firstly, G4-DOTA-Mal and Cu(II) nitrate solutions were equilibrated for 10 minutes, then the complex was added to liposomes solutions. The following samples were tested: a) 0.65 mM of lecithin + 0.728 mM/0.364 mM/0.182 mM Cu(II) + 0.04 mM of G4-DOTA-Mal; and b) 0.83 mM of DMPC or DMPC/DMPG 3% + 0.929 mM/ 0.464 mM/ 0.230 mM Cu(II) + 0.05 mM (G4-DOTA-Mal).

LEC was used at a different concentration with respect to DMPC and DMPC/DMPG 3% since the lipid concentrations were initially selected in order to optimize the structural characteristics of liposomes for performing DLS measurements. However, the molar ratios between lipids, dendrimers and Cu(II) ions were maintained constant in all experiments.

The volume vs. size plots of dendrimer alone, liposomes alone, and the samples obtained by adding dendrimers to liposomes in the absence and presence of Cu(II) at the different concentrations allowed us to evaluate the particle sizes to structurally characterize the systems, together with PDI and ZP. As a reference, the dendrimer alone was also investigated in aqueous solution (Figure S3).

4.2 EPR experiments

The suspension was placed in EPR tubes (1 mm internal diameter) and subjected to different equilibration times (t = 0, 1 and 24 h). All experiments were performed at 25 °C. Data were analyzed via Budil at al. calculation program.[Budil, D. E.et al. J. Magn. Reson., **1996**, 120, 155-189].

For the analysis of Cu(II) spectra, according to the sensitivity of the instrument, the used experimental concentrations were 25 mM for the phospholipids, 28 mM/ 14 mM/ 7 mM for Cu(II), and 1.56 mM for G4-DOTA-Mal. First, G4-DOTA-Mal and Cu(II) nitrate solutions were mixed under stirring for 10 minutes, then liposome solutions were added.

For the analysis of the spectra of the spin probe CAT12, CAT12 (1 mM) was added to the liposome solutions (25 mM in phospholipids), and the final liposomal suspension was stirred overnight.

For both the EPR studies using Cu(II) and CAT12, the EPR spectra of samples consisting of the ternary system constituted by the paramagnetic species + dendrimer + liposome were compared to the simple binary systems constituted by the paramagnetic species + liposome or dendrimer.

4.3 Fluorescence anisotropy experiments

Fluorescence spectra were measured by means of a Fluorolog 3 (Horiba JobinYvon, USA) fluorescence spectrophotometer. The polarization values (r) were calculated using FluorEssence software with the following equation:

$$\text{Anisotropy}, r = \frac{I_{VV} - GI_{VH}}{I_{VV} + 2GI_{VH}} \qquad \text{S1}$$

Where I_{VV} stands for vertical excitation and vertical emission; I_{VH} stands for vertical excitation and horizontal emission; G is the grating factor which is I_{HV}/I_{HH}. For the three different liposomes (LEC, DMPC and DMPG) the phospholipids (500 μM) were dissolved in chloroform/ methanol 2:1 in presence of the fluorescent probe, 1,6-diphenylhexatriene (DPH) or 1-(4-(trimethylamino)phenyl)-6-phenylhexa-1,3,5-triene (TMA-DPH), at a lipid/ fluorescent probe molar ratio of 500:1. The liposome preparation was performed as described above. Then, a sample of liposomes (1 ml) was placed in the semi-cuvette and 10 μL of G4-DOTA-Mal (C_{Stock} = 0.001M in MilliQ) was added to each measurement. The concentration of G4-DOTA-Mal was increased from 0 μM to 100 μM during measurements. The wavelength at which the analysis was carried out were 380 nm as excitation, 426 nm as

emission for DPH and 430 nm for TMA-DPH. All the experiments were performed at room temperature.

Fluorescence anisotropy provides information on the main localization of molecules by means of specific fluorescent probes. In our case we evaluated the membrane fluidity and glycodendrimer localization in presence of the three liposomes: LEC, DMPC and DMPG, using the following two fluorescent probes [Do Canto, A. M. T. M.et al. BBA Biomembranes, **2016**, 1858(11), 2647–2661]: 1) DPH is a popular probe of membrane interiors, useful for evaluating the possible interaction between glycodendrimer and liposomes in the hydrophobic membrane region. It provides info on mobility not on localization. In fact DPH can be between the chains but also flat in the core of the bilayer, with a not defined localization; (2) TMA-DPH is well-known to act as an anchor on membrane surface; the molar moiety limits the insertion but the DPH part is still in the upper part of the hydrophobic section. In the present study, DPH was used to detect surface interactions between glycodendrimer and liposomes surface.

4.4 Cryo-TEM images

Samples for acquiring Cryo-TEM images were prepared by dropping 2 μL of liposome solution (1.3 mM LEC + 0.08 mM DOTA in PBS 10 mM; 1.5 mM DMPC or DMPG + 0.09 mM DOTA in PBS 10 mM) on copper grids coated with holey carbon foil (so-called Lacey type). A piece of filter paper was used to remove the excess water; the sample was then rapidly frozen in liquid ethane at -178°C. The blotting with the filter paper and plunging into liquid ethane was done in a Leica GP device (Leica Microsystems GmbH, Wetzlar, Germany). All images were recorded in bright field at -172°C. The diameter (60-80 particles) and membrane thickness (10-20 particles) of the empty and loaded-liposome were determined from cryo-TEM images by using TEM Image Processing Software.

5. Additional information for the Results and Discussion

5.1 DLS and ZP study providing size and charge of dendrimers and/or liposomes in the absence and presence of Cu(II)

Figure S3 shows the volume plot of G4-DOTA-Mal (0.05 mM) in aqueous solution evaluated by dynamic light scattering experiments. D_h = hydrodynamic diameter.

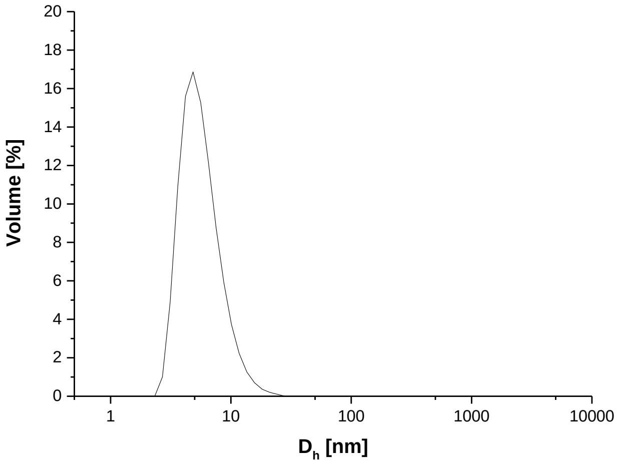

Figure S3. Volume as a function of size distribution for G4-DOTA-Mal (0.05 mM) in aqueous solution evaluated by dynamic light scattering experiments. D_h = hydrodynamic diameter.

Figure S4 shows the pH-dependence of the zeta potential of G4-DOTA-Mal.

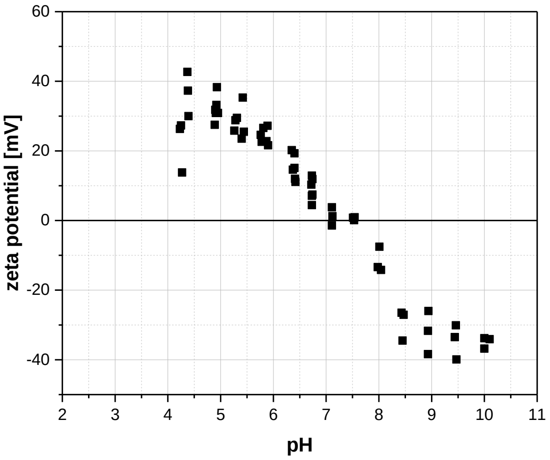

Figure S4. pH-dependent zeta potential of G4-DOTA-Mal

Table S1 summarizes the D_h, Z-average values, and surface charge (ζ) of aggregated G4-DOTA-Mal macromolecules (0.04 M) in absence and presence of Cu(II) (7, 14 and 28 mM) at 25 and 37 °C, respectively.

Table S1. Z-average (diameter, D_h), PDI and ZP (ζ) of G4-DOTA-Mal (0.05 mM) in absence and presence of Cu(II) in PBS (10 mM) at 25 and 37°C.

**Samples**	**25°C**		**37°C**		**25°C**	**37°C**
	**D_h nm**	**PDI**	**D_h nm**	**PDI**	**ζ mV**	**ζ mV**
G4-DOTA-Mal	337	0.500	670	0.564	16.5	21.5
G4-DOTA-Mal + 7 mM Cu(II)	583	0.622	1072	0.737	25.0	26.1
G4-DOTA-Mal + 14 mM Cu(II)	338	0.412	632	0.534	28.3	26.0
G4-DOTA-Mal + 28 mM Cu(II)	186	0.477	436	0.403	32.0	28.3

Z-average are shown, but the samples are highly polydisperse, the D_h data is not reliable.

Table S2 depicts the hydrodynamic diameters (D_h, Z-average) and surface charge (ζ) of liposomes (LEC, DMPC, and DMPG) in absence and presence of G4-DOTA-Mal and their adducts with Cu(II) at three concentrations (7 mM, 14 mM and 28 mM), determined at 0, 1 and 24h.

Table S2. Z-average (diameter, D_h), PDI and ZP (ζ) of the different liposomes (LEC, DMPC, DMPG) in absence and presence of G4-DOTA-Mal (0.05 mM) and Cu(II) in PBS (10 mM) at 25°C. On the basis of DLS results, ZP was only measured after the equilibration of the sample (24 h).

Sample	0 h		1 h		24 h		
**LEC**	**D_h nm**	**PDI**	**D_h nm**	**PDI**	**Dh nm**	**PDI**	**ζ mV**
Pure liposome	130	0.037	130	0.037	130	0.037	-6.37
0 mM Cu(II) + G4-DOTA-Mal	130	0.154	132	0.154	135	0.154	-1.39
7 mM Cu(II)+ G4-DOTA-Mal	255	0.430	190	0.258	130	0.190	3.80
14 mM Cu(II) + G4-DOTA-Mal	186	0.420	130	0.235	131	0.193	0.09
28 mM Cu(II) + G4-DOTA-Mal	136	0.129	169	0.347	127	0.156	2.03

	0 h		1 h		24 h		
**DMPC**	**D_h nm**	**PDI**	**D_h nm**	**PDI**	**D_h Nm**	**PDI**	**ζ mV**
Pure liposome	129	0.040	129	0.040	129	0.040	-0.32
0 mM Cu(II) + G4-DOTA-Mal	156	0.363	122	0.340	116	0.198	-0.65
7 mM Cu(II)+ G4-DOTA-Mal	191	0.403	142	0.310	132	0.313	1.21
14 mM Cu(II) + G4-DOTA-Mal	150	0.368	119	0.327	119	0.214	1.75
28 mM Cu(II) + G4-DOTA-Mal	143	0.381	121	0.375	170	0.326	2.54

	0 h		1 h		24 h		
**DMPG**	**D_h nm**	**PDI**	**D_h nm**	**PDI**	**D_h Nm**	**PDI**	**ζ mV**
Pure liposome	119	0.038	119	0.038	119	0.038	-7.20
0 mM Cu(II) + G4-DOTA-Mal	140	0.365	144	0.260	129	0.430	-1.47
7 mM Cu(II)+ G4-DOTA-Mal	137	0.284	167	0.283	113	0.319	-0.12
14 mM Cu(II) + G4-DOTA-Mal	158	0.305	124	0.277	122	0.369	0.02
28 mM Cu(II) + G4-DOTA-Mal	173	0.267	127	0.301	96	0.226	0.69

Concerning the lecithin liposomes, the post-loading addition of G4-DOTA-Mal had no effect on the liposome size. The addition of glycodendrimers is reflected in immediate increase in PDI followed by restoration of initial conditions after 24 hours (PDI lower than 0.2, resulting in homogenous systems). Additionally, the interaction of the G4-DOTA-Mal - Cu(II) complex and LEC resulted in a further increase in the positively charged surface and the system appeared homogenous at t=0h, t=1h and t=24h.

The interaction between the G4-DOTA-Mal - Cu(II) complex and DMPC liposomes makes the surface charge positive. Unlike what was observed for lecithin liposomes, for DMPC systems the Cu(II) concentration was less affecting the polydispersity of the particles.

For DMPG (DMPC/DMPG 3 %) liposomes, as shown in Table S2, the addition of G4-DOTA-Mal and the further addition of Cu(II) to the dendrimer increases the positive charge of the DMPG surface. Concerning the polydispersity, the presence of G4-DOTA-Mal and the complex is reflected in the increase of PDI also after 24 h.

The volume in function of the size distribution in Figure S5 shows a peak around 100 nm highlighting that no aggregation processes occurred. The hydrodynamic diameter obtained from DLS refers to particles diffusion within a fluid; surface structure, concentration as well as the type of ions in the medium affect the diffusion speed of particles and, consequently, the hydrodynamic diameter.

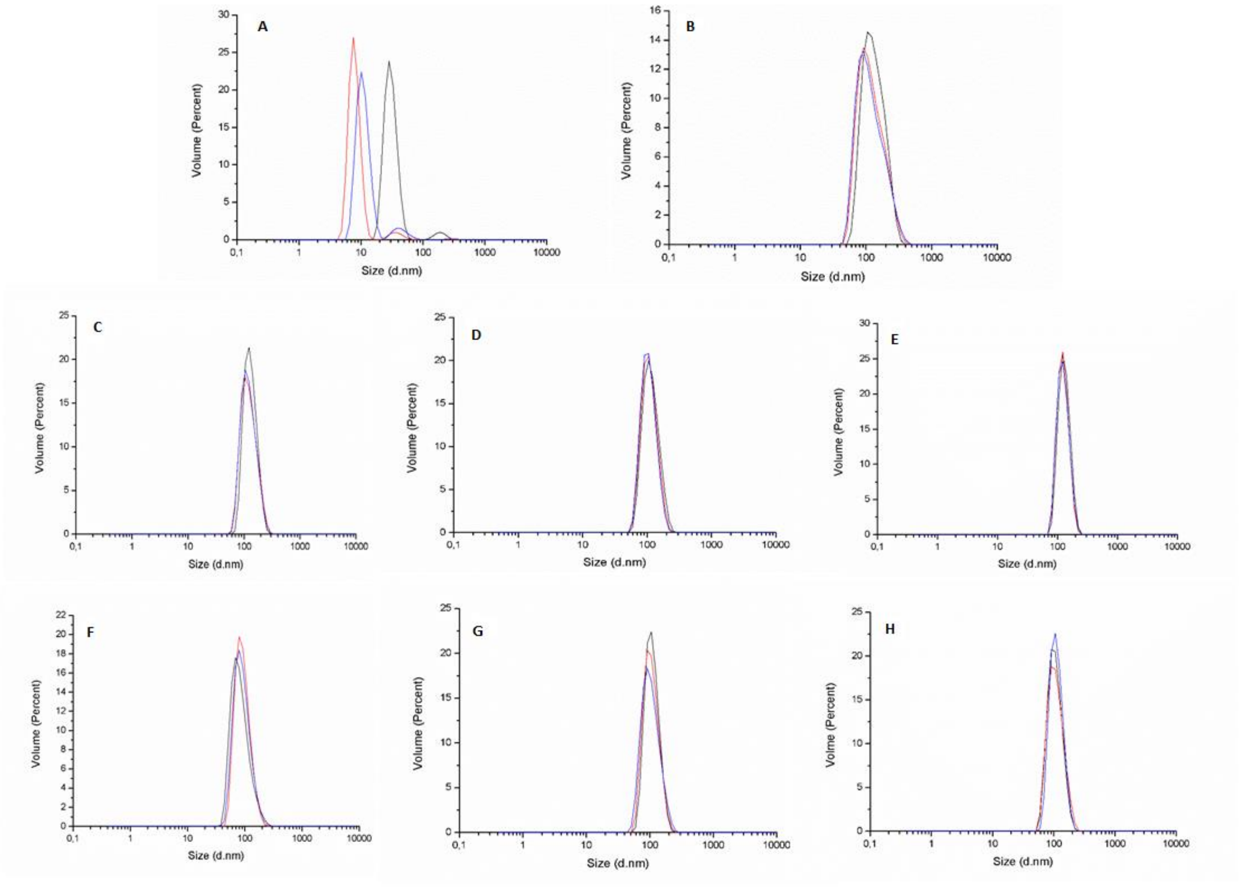

Figure S5. DLS volume plot of G4-DOTA-Mal (A) showing 3 measurements of the same batch in PBS (10 mM). DLS volume plots for LEC liposomes + G4-DOTA-Mal (B); DMPC liposomes (C); DMPC liposomes and G4-DOTA-Mal (D); DMPC liposomes, G4-DOTA-Mal and Cu(II) (E); DMPG liposomes (F); DMPG liposomes and G4-DOTA-Mal (G); DMPG liposomes, G4-DOTA-Mal and Cu(II) (H). All compounds were dissolved in PBS (10 mM, pH 7.4) at 25°C; compounds concentrations reported in the experimental section. 3 measurements of the same batch in PBS (10 mM) for B to H.

Therefore, a well-defined liposome structure forms both in the absence and in the presence of the glycodendrimer and Cu(II).

5.2 Long-term stability of liposomes at different temperatures by DLS

Further DLS measurements over time were performed to verify the stability of the liposomes (Figures S6-S8). Figure S6 shows the difference between LEC liposomes obtained using sonication or extrusion treatments. The sonication treatment (Figure S6) leads to unstable and non-homogenous liposomes; the system is indeed characterized by a high polydispersity index (PDI greater than 0.2; results not shown). Conversely, the extrusion treatment produces homogeneous, monolamellar and long-term stable liposomes.

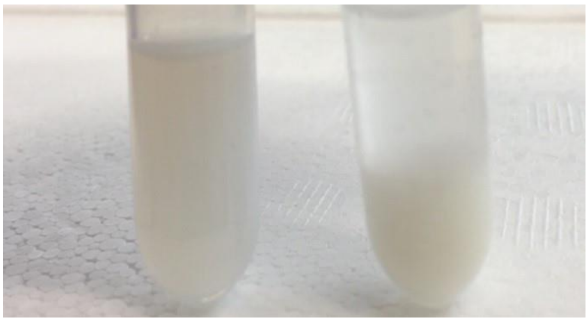

Figure S6. Lecithin suspension after the overnight incubation: on the left, the sample obtained by extrusion treatment, on the right, the sonication one.

Figures S7 and S8 show that the variation of the average size of liposomes obtained by means of the extrusion method at 25 °C and 37 °C, respectively, was almost constant throughout one month; in particular, there was a good similarity between the liposomes stored at room temperature and those at 4 °C.

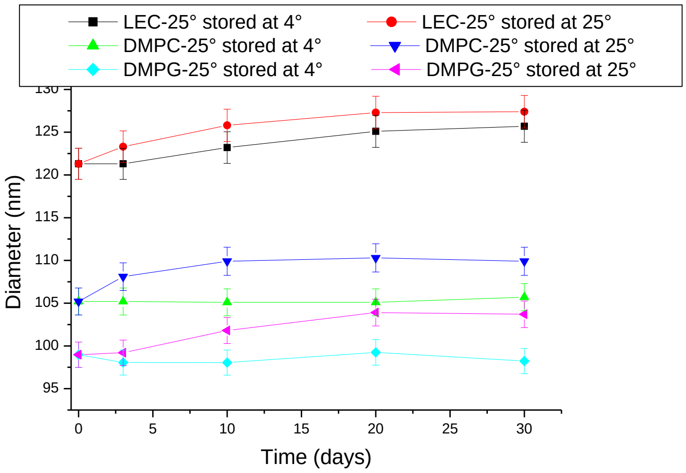

Figure S7. Variation of the liposome diameter (mean z-average diameter) over equilibration time at 25 °C, proving the long-term stability of the liposomes obtained by the extrusion method at different temperatures (4 and 25 °C). The liposomes were stored at 4ºC or 25ºC for different periods (3, 10, 20 and 30 days). The DLS was carried out at 25ºC. C_{LEC} = 0.5 mg/mL; C_{DMPC} = 0.3 mg/mL; C_{DMPG} = 0.3 mg/mL. Data are expressed as mean ± SD.

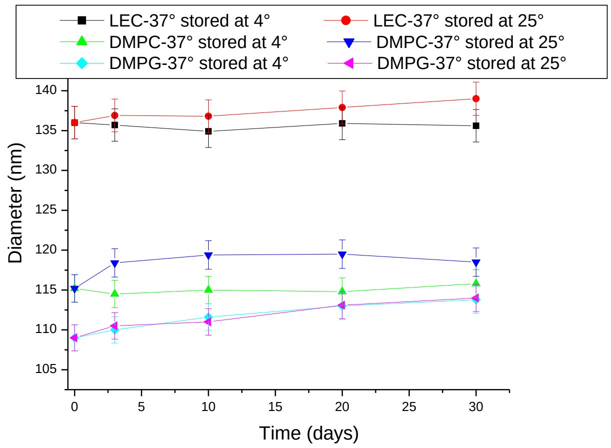

Figure S8. Variation of the liposome diameter (mean z-average diameter) over equilibration time at 37 °C, proving the long-term stability of the liposomes obtained by the extrusion method at different temperatures (4 and 25 °C). The liposomes were stored at 4ºC or 25ºC for different periods (3, 10, 20 and 30 days). The DLS was carried out at 37 ºC. C_{LEC} = 0.5 mg/mL; C_{DMPC} = 0.3 mg/mL; C_{DMPG} = 0.3 mg/mL. Data are expressed as mean ± SD.

Furthermore, passing from 25 °C to 37 °C the average size progressively increased for liposomes stored at room temperature as well as 4°C, due to the faster dynamics at the higher temperature.

However, the size of the liposomes was completely maintained after 30 days from samples preparation. An important conclusion is that the used protocol, associated with the extrusion treatment, leads to the production of unilamellar liposomes that are stable and uniform over time, showing PDI values lower than 0.2 and having reproducible diameters. In addition to this, the liposomes show to be resistant to temperature changes.

Furthermore, the observations confirm that the size of the vesicles are not concentration and temperature dependent for the different types of lipids.

5.3 Further examples of CAT12 EPR spectra

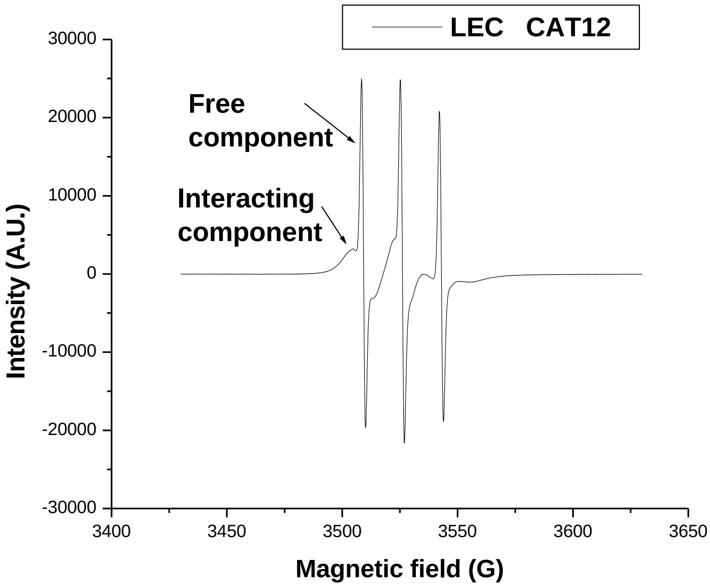

Figure S9. EPR experimental spectrum of CAT 12 in the two component system LEC/CAT12.

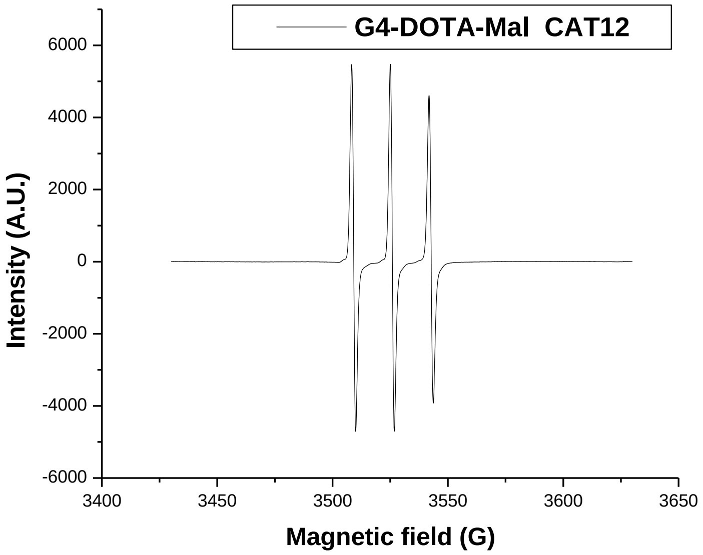

Figure S10. EPR experimental spectrum of CAT 12 in the two component system G4-DOTA-Mal/CAT12.

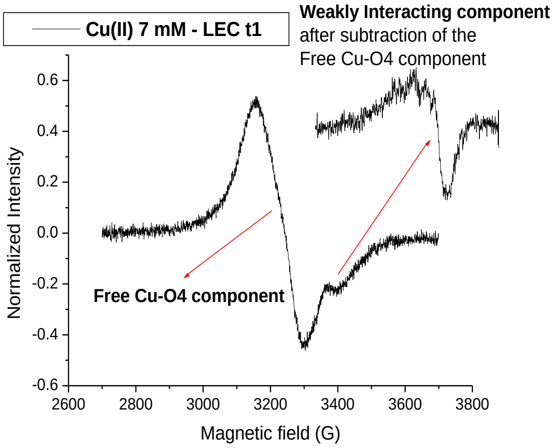

Figure S11. EPR spectrum of the two component system LEC/Cu(II) with a Cu(II) concentration of 7 mM at 1 h.

5.4 Further comments on the variations of the EPR spectra intensity, relative % of the interacting component, τ for the Free component, S of the interacting component, <A> for the Interacting and Free components, for the three different liposomes in the absence and presence of G4-DOTA-Mal (shown in Figure 4 of the manuscript):

The **intensity** increases in the presence of liposomes compared to pure CAT12 solutions, confirming that CAT12 inserts into the liposomes (LEC < DMPC < DMPG) increasing its solubility.

The lower solubility found for LEC compared to other liposomes was accompanied by a higher relative **percentage of interacting component**. By adding G4-DOTA-Mal, the interacting percentage increased (DMPC<DMPG<LEC).

The **microviscosity measured by τ for the free component** followed the series (DMPC<DMPG<LEC) in the absence of the dendrimer, which become (pure CAT12<DMPG<DMPC<LEC) in presence of the dendrimer. This behavior reflects the interactions occurring at the dendrimer/liposome surface, connected to the presence of the hydrocarbon chain in CAT12, which, in the free component, stays at the interface and not inside the liposome. Therefore, by adding G4-DOTA-Mal, the microviscosity of the free component increases, because the dendrimer interacts with the liposome surface and blocks CAT12. This increase is more significant for DMPC with respect to the other liposomes as a consequence of the more compact DMPC structure. For DMPG the presence of the charge makes this increase less significant.

A minor internalization of CAT12 in compact DMPC liposomes was proved by the lower value of the **order parameter S** (DMPC<LEC<DMPG). S decreased by adding G4-DOTA-Mal to all liposomes, in line with a perturbative effect due to liposome-dendrimer interactions.. This decrease was more significant for DMPG, which reached the value of LEC. Therefore, the DMPG liposome structure was partially deformed due to the interaction with the dendrimer.

The variation of the **polarity parameter, <A> for the interacting probes** follows the trend DMPG<DMPC<LEC. The lower micropolarity for DMPG is probably due to neutralization of the positive charge of the CAT group with the negative charge of the liposome surface

The micropolarity <A> of the free component (DMPC ~ LEC<DMPG) varied in an opposite way if compared to the interacting component: in fact <A> was the higher for DMPG (due to the negative charge) and increased from the absence to the presence of G4-DOTA-Mal in agreement with a location of the radical group at the external surface of the

liposomes where the charged groups of the phospholipids are located. G4-DOTA-Mal created a more polar environment for CAT12 surfactants at the liposome/dendrimer interface.

5.5 Fluorescence anisotropy study

Fluorescence anisotropy provides information on the main localization of molecules by means of specific fluorescent probes. In our case we evaluated the membrane fluidity and glycodendrimer localization in presence of the three liposomes: LEC, DMPC and DMPG, using two fluorescent probes: (1) DPH is a popular probe of membrane interiors, useful for evaluating the possible interaction between glycodendrimer and liposomes in the hydrophobic membrane region; (2) TMA-DPH is well-known to act as an anchor on membrane surface; in the present study, it was used to detect surface interactions between glycodendrimer and liposomes. The change in the fluorescence anisotropy is shown in Figure S9 as a function of the dendrimer concentration towards the three liposomes, LEC, DMPC and DMPG.

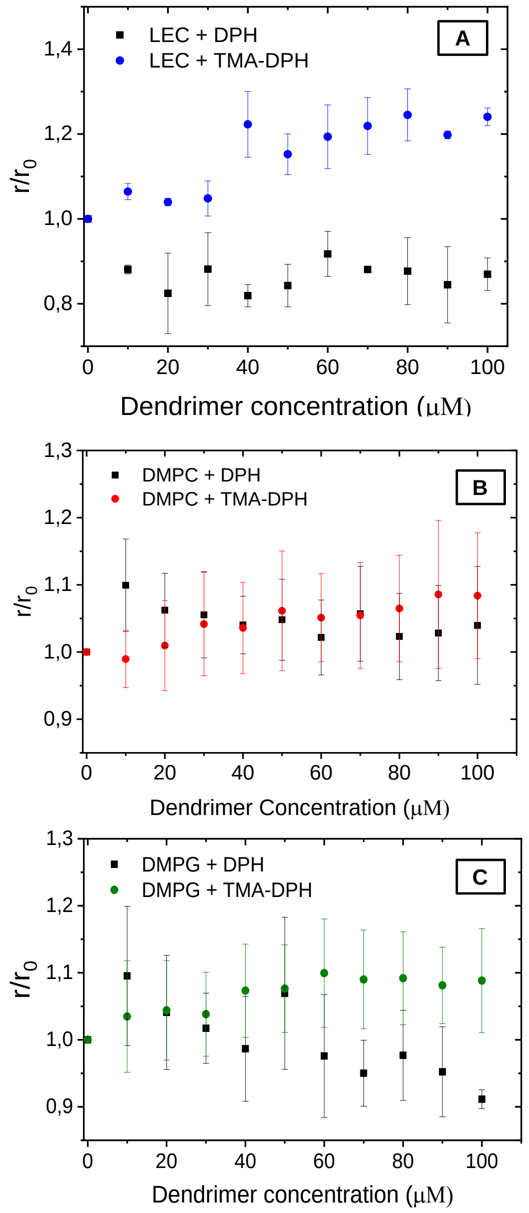

Figure S12. Fluorescence anisotropy of DPH and TMA-DPH probes in LEC (A), DMPC (B) and DMPG(C) liposome/G4-DOTA-Mal mixtures. r, fluorescence anisotropy value; r0 control fluorescence anisotropy value in the absence of G4-DOTA-Mal. Data are expressed as mean ± SD.

A more decisive hydrophilic interaction of TMA-DPH with the glycodendrimer is shown by LEC (Figure S9, top), if compared to the other liposomes, DMPC and DMPG (Figure S9,

middle + bottom). This implies a surface interaction of G4-DOTA-Mal with LEC, which is in line with the EPR results. Weaker interactions are present in case of liposomes, DMPC and DMPG, with nearly differences. The hydrophilic surface interactions of TMA-DPH-integrated liposomes with G4-DOTA-Mal are only visible at higher dendrimer concentration. This is partially explainable, when considering the surface charge (Table S2) and perturbation properties of liposomses, induced by G4-DOTA-Mal (EPR study), only a combination of both parameters may induce hydrophilic interaction of less extent with G4-DOTA-Mal.

Using the hydrophobic probe DPH, negligible interactions are observed in all samples (Figure S9). In the case of DMPG at low dendrimer concentration, a small interaction occurs with the hydrophobic part of the membrane probably related to the structural deformation hypothesized on the basis of the EPR results. As the concentration increases, hydrophilic interactions prevail.
